# Remote Actuation of Magnetic Nanoparticles For Cancer Cell Selective Treatment Through Cytoskeletal Disruption

**DOI:** 10.1038/srep33560

**Published:** 2016-09-20

**Authors:** Alyssa M. Master, Philise N. Williams, Nikorn Pothayee, Nipon Pothayee, Rui Zhang, Hemant M. Vishwasrao, Yuri I. Golovin, Judy S. Riffle, Marina Sokolsky, Alexander V. Kabanov

**Affiliations:** 1 Center for Nanotechnology in Drug Delivery, University of North Carolina, Chapel Hill, NC, USA; 2 Department of Pharmaceutical Sciences, University of Nebraska Medical Center, Omaha, NE, USA; 3 Macromolecules and Interfaces Institute, Virginia Polytechnic Institute and State University, Blacksburg, VA, USA; 4 Nanocenter, G. R. Derzhavin Tambov State University, Tambov, 392000, Russian Federation; 5 Laboratory of Chemical Design of Bionanomaterials, Faculty of Chemistry, M. V. Lomonosov Moscow State University, Moscow, 117234, Russian Federation

## Abstract

Motion of micron and sub-micron size magnetic particles in alternating magnetic fields can activate mechanosensitive cellular functions or physically destruct cancer cells. However, such effects are usually observed with relatively large magnetic particles (>250 nm) that would be difficult if at all possible to deliver to remote sites in the body to treat disease. Here we show a completely new mechanism of selective toxicity of superparamagnetic nanoparticles (SMNP) of 7 to 8 nm in diameter to cancer cells. These particles are coated by block copolymers, which facilitates their entry into the cells and clustering in the lysosomes, where they are then magneto-mechanically actuated by remotely applied alternating current (AC) magnetic fields of very low frequency (50 Hz). Such fields and treatments are safe for surrounding tissues but produce cytoskeletal disruption and subsequent death of cancer cells while leaving healthy cells intact.

The medicines of the future should be dormant on the way to their target but actuated to execute their therapeutic function once they reach the site of their action within the body. Superparamagnetic iron oxide nanoparticles (SMNP) can be remotely actuated by externally applied magnetic fields to kill cancer cells^1^,^2^,^3^,^4^. One of the most studied modes of remote actuation is magnetic hyperthermia, which utilizes the iron oxide particle response to alternating current (AC) magnetic fields of relatively high frequencies, on the order of hundreds of kHz. Once exposed to such fields the particles generate heat through Neél or Brownian relaxation, depending on the particle and the surrounding media characteristics^5^,^6^,^7^,^8^. The heat leads to temperature increases causing subsequent damage to the surrounding cells. However, magnetic hyperthermia is limited due to challenges in synthesizing non-toxic SMNPs with sufficiently high specific absorption rates (SAR), in reaching sufficient intracellular SMNP concentrations and in restricting heat dissipation from a tumor to adjacent healthy tissues^5^. It is also clear that the thermal conductivity of water is so high that bulk temperature increase is difficult. In response to the issue of the high thermal conductivity of water, the concept of surface heating has been proposed. This concept emphasizes energy dissipation in the absence of measurable bulk heating, and suggests that localized surface heating may be the cause of cell death.

Several studies now document cell damage with exposure to AC magnetic fields even without a perceptible increase in temperature^9^,^10^,^11^,^12^,^13^,^14^. For example, Villanueva *et al*. reported HeLa tumor cell damage and death after incubation with magnetic manganese oxide nanoparticles that were coated with silica, and exposure to an AC field with a frequency of 10 Hz^9^. Rinaldi *et al*. incubated magnetic iron oxide nanoparticles that were coated with carboxymethyldextran and conjugated with epidermal growth factor (EGF) with MDA-MB-468 cancer cells that have EGFR receptors^10^,^11^. Upon exposure to an AC field with a strength of 30.5 kA/m and 233 kHz, cell viability was dramatically reduced without any measured temperature rise from 37 °C^10^. Sanchez and Connord *et al*. designed and constructed a magnetic field space within a confocal microscope, and viewed cell behavior dynamically upon exposure to an AC field of 53 mT at 300 kHz^12^,^13^. Iron oxide particles with an average diameter of 8.7 nm were coated with targeting ligands that facilitated their selective uptake into endocrine cancer cells. Cell responses included lysosome membrane permeabilization with concomitant ROS appearance, followed by cell death, and the responses were largely limited to cells that contained the nanoparticles and also that were exposed to the AC field. Importantly, this suggests that targeted cells could be made to respond to the field without damage to neighboring cells.

Magneto-mechanical actuation of cells or organelles within cells has also been suggested. For example, Zhang *et al*. designed a rotating AC magnetic field space with a field strength of ~30 mT and exposed cells at a very low frequency of ~20 Hz^14^. They utilized the field to enhance uptake of iron oxide nanoparticles of 100 nm and above that were conjugated with antibodies for targeting the lysosomal protein marker LAMP-1 Into rat insulinoma tumor cells and human pancreatic cells. They reported intracellular lysosomal membrane permeabilization followed by apoptosis, and proposed that mechanical rotation of the nanoparticles associated with the lysosomal membranes caused membrane disruption. Kim *et al*. reported utilizing relatively large nanoparticles comprised of a 20/80% Fe/Ni alloy coated with gold in a disc geometry (~60-nm thick and ~1-μm in diameter). The gold surfaces of the discs were functionalized with anti-human-IL13α2R antibodies to target the cell membranes of human glioblastoma cells. They incubated the particles with the cells and exposed them to uniform AC magnetic fields with very low strengths (~8 kA/m) and frequencies (10–20 Hz). The glioblastoma cells underwent apoptosis and it was hypothesized that the discs aligned in the field and then somewhat misaligned when the field was changed, thus damaging the cell membranes that they were bound to, and further causing in an ionic signal that resulted in cell apoptosis^15^.

Distinct from these studies, here we show a novel magneto-mechanical mechanism of action of small polymer coated SMNPs actuated inside the cells, also by super low frequency AC magnetic fields as discussed in previous reports of magneto-mechanical actuation^14^,^15^. Such fields are not expected to cause any damage to biological tissues but they result in magneto-mechanical actuation of the SMNPs and promote cancer cell death. We demonstrate cancer cell selectivity due to the intrinsic difference in cell architecture between cancerous and healthy cells. Previously, we have shown that the activity and conformation of enzymes immobilized on SMNPs were disrupted following exposure to super low frequency AC magnetic fields in a non-heat induced manner^16^. These changes in the enzyme structure were attributed to the motion of SMNPs in the AC magnetic field, which created shear and tensile forces on the surrounding materials. In this work, we demonstrate the concept of magneto-mechanical actuation of small ~7–8 nm diameter magnetite (Fe_3_O_4_) SMNPs in AC magnetic fields and their effects on the subcellular compartments of cancerous *vs* non-cancerous cell lines. We show that a non-targeted polymer coated SMNP system is taken up into cell lysosomal compartments and after magnetic field actuation, can cause cytoskeletal disruption in cancer cells while leaving healthy cells intact and viable.

## Materials and Methods

### Cell lines

MDA-MB-231 (human triple negative (ER/PR- Her2/neu-) mammary gland adenocarcinoma), BT474 (human breast ductal carcinoma) and MCF10A (human non tumorigenic mammary gland cells) were supplied by ATCC (Manassas, VA). MDA-MB-231 and BT474 cells were maintained in DMEM (high glucose) containing 10% heat inactivated FBS and 1% penicillin/streptomycin. MCF10A cells were maintained in DMEM/F12 media containing 10% heat inactivated FBS, 1% penicillin/streptomycin, 10 μL/mL human insulin and 10 ng/mL human epidermal growth factor. All cell cultures were maintained at 37 **°**C in a 5% CO_2_ atmosphere. Human breast cancer cell models were used for this study. MDA-MB-231 human breast cancer cells were initially used to assess the ability of this system to kill a triple negative (ER-/PR-/HER2/neu-) cancer. BT474 human breast ductal carcinoma cells were used to further assess the effects in a cell line with a different cytoskeletal structure. Lastly, MCF10A nontumorigenic human breast cells were used as a control.

### Materials

Lysotracker^®^ Green, TubulinTracker™, Hoechst 33342, Annexin V, Propidium Iodide, fetal bovine serum (FBS) (both dialyzed and heat inactivated), Dulbecco’s Modified Eagle’s Medium (DMEM), DMEM:F-12, penicillin/streptomycin, human insulin, human epidermal growth factor and Alexa Fluor 647-hydrazine were purchased from Life Technologies (Carlsbad, CA). Hydrogen peroxide was purchased from Thermo Fisher Scientific (Waltham, MA). Lab-Tek II Chambered Coverglass #1.5 Borosilicate 8 well chambers, used for live cell imaging, were purchased from Fisher Scientific (Waltham, MA). High binding strip plates (2 × 8 MICROLON 96 well) were purchased from Griener Bio-One. MTT reagent (3-(4,5-dimethylthiazol-2-yl)-2,5-diphenyltetrazolium bromide) was purchased from Research Products International (Prospect, IL). Cytochalasin D (CD), dimethylsulfoxide (DMSO) and nitric acid (HNO_3_) TRACESELECT purity grade, Atto 647 *N*-hydroxysuccinimide ester, and Sephadex G-50 were purchased from Sigma Aldrich (St. Louis, MO). Pluronic^®^ P85, poly(ethylene oxide)_26_-b-poly(propylene oxide)_39_-b-poly(ethylene oxide)_26_ block copolymer was provided by BASF Corp. (Wyandotte, MI). PAA-*b*-P85-*b*-PAA pentablock copolymer was synthesised as shown in Supplementary Figure S1 and described in Supplementary Methods and Results online. All other chemicals were of reagent grade and used without further purification.

### Alternating Current Magnetic Field Generator

The super-low frequency alternating current (AC) magnetic field generator was custom designed and purchased from Nanomaterials Ltd. (Tambov, Russia). The unit contains a sinusoidal current generator with variable power (up to 1.5 kW), frequency (in the range from 30 to 3000 Hz) and variable magnetic field amplitude (from 10 to 100 mT). The unit is equipped with a water-cooled inductor with a ferromagnetic core and a temperature-controlled cuvette. The temperature-controlled holder accommodates one 8-well strip plate at a time. The temperature was maintained at 37 °C for all cellular experiments. For all cell experiments, cells were seeded in the middle wells, which were exposed to a homogeneous field. The experiments were conducted at a frequency of 50 Hz and the magnetic field intensity was 50 or 100 kA/m. Field frequency and field intensity were measured and monitored by an oscilloscope throughout the application time.

#### Synthesis and Characterization of Polymer-SMNP Complexes

The SMNP complexes were prepared and coated by ligand exchange with polyanion-PEG or polyanion-P85 block copolymers as described in Supplementary Methods and Results online. The effective hydrodynamic diameter (D_i_ = intensity Z-average), polydispersity and ζ-potential of the polymer-SMNP complexes were determined by dynamic light scattering (DLS) using a Zetasizer Nano ZS (Malvern Instruments Ltd., Malvern, UK). All measurements were performed in automatic mode at 25 °C. All measurements were performed at least in triplicate to calculate mean values ± standard deviations.

The polymer content in the polymer-SMNP complexes was determined by thermogravimetric analysis (Q50, TA Instruments, New Castle, DE). Approximately 10–15 mg of the samples were loaded and exposed to a heat ramp to 110 °C at a rate of 10 °C/min, followed by an isothermal hold for 15 min, and then continued heating to 1000 °C at 10 °C/min. Iron content in the polymer-SMNP complexes was analyzed by Inductively Coupled Plasma Mass Spectrometry (ICP-MS) (NexION 300D, Perkin Elmer, Waltham, MA). Briefly, 0.5 mL of particle solution (1 mg/mL) were mixed with 50 μL of nitric acid and incubated at 70 °C for at least 12 h. Following the digestion, the volume of the solution was adjusted to 1 mL with deionized (DI) water and analyzed by ICP-MS.

### Labeling of PAA-P85-SMNP with Alexa Fluor^®^647

PAA-P85 coated SMNP complexes were labeled with the fluorescent dye Alexa-Fluor^**®**^647 hydrazine using standard EDC chemistry. Briefly, 4.5 mg of PAA-P85-SMNPs were diluted with 0.35 mL of DI water and sonicated for 30 min followed by addition of 10 mg of 1-ethyl-3-(-3-dimethylaminopropyl) carbodiimide hydrochloride (EDC). A stock solution of *N*-hydroxysulfosuccinimide (S-NHS) (40 mg/mL in DI water) was prepared and 50 μL of this solution was added to the reaction vial. A stock solution of Alexa Fluor^**®**^647 hydrazine (1 mg/mL in DI water) was prepared and 0.1 mL was added to the reaction vial. The vial was protected from light and incubated overnight on a shaker at approximately 100 rpm. Alexa Fluor^**®**^647-PAA-P85-SMNPs were purified on a size exclusion column (Sephadex G-50) with PBS as the eluent followed by centrifugal filtration with 100 kDa cutoff Centricons (EMD Millipore, Billerica, MA). The concentration of SMNPs in solution was determined by ICP-MS. Similar to the previously described method, 20 μL of particle solution were mixed with 50 μL of nitric acid and incubated at 70 °C for a minimum of 12 h. Following the digestion, the volume of the solution was adjusted to 1 mL with DI water and analyzed by ICP-MS.

### Cytotoxicity of Polymer-SMNP Complexes

*In vitro* cytotoxicity of polymer-SMNP complexes was assessed in MDA-MB-231, BT474 and MCF10A cells by standard MTT assay. Briefly, cells were seeded at 5 × 10^3^ cells/well in a 96-well plate and were allowed to adhere for two days. Cells were treated with polymer-SMNP complexes at various doses (0.005–0.5 mg/mL polymer-SMNP complexes) for 24 h at 37 °C, washed with acidic saline (pH 3) to remove non-internalized polymer-SMNPs and maintained in complete DMEM for an additional 24 h. All of the samples were tested in triplicate. A standard MTT assay was then performed by addition of 25 μL of 3-(4,5-dimethylthiazol-2-yl)-2,5-diphenyltetrazolium bromide (MTT) dye (5 mg/mL) to each well followed by a 4-h incubation period at 37 °C. The resultant formazan was then solubilized in dimethylsulfoxide (DMSO) and absorption was measured at 570 nm using a spectrofluorometer (SpectraMax M5, Molecular Devices Co., USA). The reading taken from the wells with cells cultured with control medium was used as a 100% viability value. The cell viability was calculated as A_sample_/A_control_ × 100%.

### Quantitative Uptake of Polymer-SMNP Complexes *In Vitro*

MDA-MB-231, BT474 and MCF10A cells were seeded at 1 × 10^6^ cells/well in 6 well plates and allowed to adhere for 3 days. They were then washed and treated with polymer-SMNP complexes at various doses (0.005–0.5 mg/mL polymer-SMNP complexes) for 1 h or 24 h at 37 °C. Cells were rinsed 3 times with acidic saline (pH 3) and harvested using 0.05% trypsin/EDTA. Cells were pelleted, the supernatant was discarded and the cells were re-suspended in 0.5 mL of DI water. The cell suspension was then sonicated with a probe sonicator at 10 kHz for 40 s. The cell suspension was digested using nitric acid as previously described. Following the digestion, the volume of the solution was adjusted to 1 mL with DI water and analyzed by ICP-MS.

### Intracellular Distributions of PAA-P85-SMNPs

MDA-MB-231, BT474 and MCF10A cells were seeded at 1 × 10^5^ cells/well in 8-well Lab-Tek II Chamber slides. Cells were allowed to adhere for 3 days and were treated with a specified dosage of Alexa Fluor^**®**^647-PAA-P85-SMNP for 24 h. After thorough washing, the cells were treated with 100 nM of Lysotracker™ Green (**λ**_ex_/**λ**_em_ = 504/511 nm) for 1 h and Hoechst 33342 nuclear stain for 15 min. Cells were washed 3X with PBS and kept in complete media for imaging. Live cell images were acquired using a Zeiss CLSM 710 Spectral Confocal Laser Scanning Microscope with the 63X/1.4 Oil Plan Apo lens. Lysotracker™ and SMNP colocalization was determined using the Colocalization Threshold tool in ImageJ/Fiji (NIH, Bethesda, MD).

For transmission electron microscopy (TEM), cell monolayers were grown on Thermanox plastic substrates. The cells were treated with 0.1 mg/mL polymer-SMNPs for 24 h. Post-treatment, the cells were washed with PBS and fixed in 2% paraformaldehyde/2.5% glutaraldehyde/0.15 M sodium phosphate buffer, pH 7.4, for 1 h at room temperature and stored at 4 °C until processed. Following 3 rinses with 0.15 M sodium phosphate buffer, pH 7.4, the cells were post-fixed with 1% osmium tetroxide/0.15 M sodium phosphate buffer for 1 h at RT. After washes in DI water, the cells were dehydrated using increasing concentrations of ethanol (30%, 50%, 75%, 100%, 10 min each) and embedded in Polybed 812 epoxy resin (Polysciences, Inc., Warrington, PA). The cells were sectioned *en face* to the substrate at 70 nm using a diamond knife. Ultrathin sections were collected on 200 mesh copper grids and stained with 4% aqueous uranyl acetate for 15 min, followed by Reynolds’ lead citrate for 7 min^17^. Samples were viewed with a LEO EM910 transmission electron microscope (Carl Zeiss Microscopy, LLC, Peabody, MA) with an acceleration voltage of 80 kV. Digital images were taken using a Gatan Orius SC 1000 CCD Camera and DigitalMicrograph 3.11.0 software (Gatan, Inc., Pleasanton, CA).

### Effect of Exposure to AC Magnetic Fields on Cell Viability

MDA-MB-231, BT474 and MCF10A cells were seeded at 5 × 10^3^ cells/well in 2 × 8 MICROLON 96 well high binding plate strips (Griener Bio Inc.) and were allowed to adhere for 3 d. Cells were treated with PAA-P85-SMNP complexes at various concentrations (0.05–0.5 mg/mL polymer-SMNP complexes) for 24 h at 37 °C, washed with acidic saline (pH 3) and exposed to AC magnetic fields of 50 or 100 kA/m and 50 Hz as specified in the legends. In the continuous mode, the cells were exposed to the field for 30 min. In the pulsed exposure mode, the cells were exposed to the field with a 10 min on, 5 min off pattern for 30 min of the field being on in total. During the experiments the temperature was maintained at 37 °C. All the samples were tested in triplicate. A standard MTT assay was then performed.

### Intracellular Distributions of PAA-P85-SMNP Complexes After Exposure to an AC Field

MDA-MB-231, BT474 and MCF10A cells were seeded at 1 × 10^5^ cells/well in 8-well Lab-Tek II Chamber slides. Cells were allowed to adhere for several days and were treated with a specified dosage of Alexa Fluor^**®**^647-PAA-P85-SMNPs. After 24 h, the cells were washed and then exposed to an AC magnetic field (50 Hz, 50 kA/m) using the pulsed exposure regime for a total of 30 min (i.e., 10 min on, 5 min off, for a total of 30 min on). Twenty four hours post exposure, the cells were treated with 100 nM Lysotracker™ Green (**λ**_ex_/**λ**_em_ = 504/511 nm) for 1 h and Hoechst 33342 nuclear stain for 15 min. Cells were washed 3X with PBS and kept in complete media for imaging. Live cell images were acquired using a Zeiss CLSM 710 Spectral Confocal Laser Scanning Microscope with the 63X/1.4 Oil Plan Apo lens.

### Assessment of Lysosomal Membrane Permeabilization

MDA-MB-231, BT474 and MCF10A cells were seeded at 1 × 10^5^ cells/well in 8-well Lab-Tek II Chamber slides. Cells were allowed to adhere for several days and were treated with PAA-P85-SMNPs at a concentration of 0.1 mg/mL. After 24 h, the cells were washed and then exposed to an AC magnetic field (50 Hz, 50 kA/m) using the pulsed exposure regime for a total of 30 min. Three hours post exposure, cells were treated for 15 min with 10 μg/mL acridine orange stain. The cells were washed 3X with PBS and kept in complete media for imaging. Positive control cells were treated with 150 μM hydrogen peroxide for 3 h followed by thorough washing and staining with acridine orange. Live cell images were acquired using a Zeiss CLSM 710 Spectral Confocal Laser Scanning Microscope with the 63X/1.4 Oil Plan Apo lens.

### Effect of Cytoskeleton Modulation on the Response to an AC Magnetic Field

For cell viability studies, MDA-MB-231, BT474 and MCF10A cells were seeded at 5 × 10^3^ cells per well in 2 × 8 96-well high binding strip plates (Griener Bio Inc.) and were allowed to adhere for 2 d. The cells were treated with PAA-P85-SMNPs at various doses for 24 h at 37 °C followed by washing with acidic saline. After washing to remove non-internalized polymer-SMNP complexes, test cells were exposed to a 100-nM sub-lethal dosage of cytochalasin D (CD) for 1 h. After washing, the cells were exposed to the AC magnetic field and viability was tested 24 h post exposure using a MTT assay as previously described. Appropriate controls of cells exposed to just one of the compounds (either PAA-P85-SMNP alone or CD alone) as well as cells without field exposure were used.

For confocal studies, MDA-MB-231, BT474 and MCF10A cells were plated on Lab-Tek II Chamber slides at a concentration of 1 × 10^5^ cells/well and allowed to grow overnight. The cells were then treated with 0.1 mg/mL Alexa Fluor^**®**^647-PAA-P85-SMNP for 24 h followed by thorough washing with acid saline and replacement with complete media. The cells were incubated with 100-nM CD for 1 h to enact cytoskeletal damage in a nonlethal capacity. After washing, the cells were exposed to the pulsed AC magnetic field (50 Hz, 50 kA/m) (10 min on, 5 min off, total exposure 30 min on). Appropriate controls included cells not exposed to the magnetic field and untreated cells. Cells were then incubated at 37 °C for 24 h, fixed using 4% paraformaldehyde and permeabilized using 0.5% Triton-X 100. Fixed cells were stained with ActinGreen 488 (Life Technologies, Carlsbad, CA), a phalloidin-based actin stain and Hoechst 33342. Images were acquired using a Zeiss CLSM 710 Spectral Confocal Laser Scanning Microscope with the 63X/1.4 Oil Plan Apo lens.

### Statistical Analysis

Statistical analyses were performed using GraphPad Prism (GraphPad Software, Inc, La Jolla, CA). ANOVA or two-tailed Student’s t-tests was used to analyze data. Where applicable, reported p-values have been adjusted for multiple comparisons using the Ryan-Einot-Gabriel-Welsch post-hoc method. Significance was reported for p < 0.05.

## Results

### Quantitative Intracellular Uptake of Polymer-SMNP Complexes

A series of block copolymers with a polyanion block and poly(ethylene glycol) (PEG) was synthesized to evaluate the effect of polymer coating composition on the polymer-SMNP complexes’ uptake in cancer cells. The polyanion block was either polyacrylic acid (PAA) or polymethacrylic acid (PMA), which differ in their hydrophobicity. The more hydrophobic PMA was expected to interact better with the hydrophobic cell membrane and improve particle uptake. Another strategy to improve the internalization of polymer coated SMNPs was incorporation of Pluronic P85 (P85) into the polymer coating. P85 effectively accumulated in the cells across all the cell lines tested as was analyzed by flow cytometry (Supplementary Fig. S2) and confocal microscopy (Supplementary Fig. S3). Representative confocal microscopy images of BT474 and MDA-MB-231 indicate that in both cell lines P85 preferentially accumulates in lysosomes (see Supplementary Fig. S3). Due to this favorable uptake pattern, P85 was incorporated in the polymer coatings of several of our tested SMNP-complexes by complexation of SMNPs with a PAA-*b*-P85-*b*-PAA pentablock copolymer. The physicochemical characteristics of the formed polymer-SMNP complexes are summarized in Table S1. The intensity average hydrodynamic diameters (D_i_) of the polymer-SMNP complexes were in the range of 30–70 nm with ζ-potential values of −35 to −50 mV. The polymer content in all the complexes was around 60 wt% as measured by thermogravimetric analysis (TGA), and this was in excellent agreement with the iron concentration measured by inductively coupled plasma-mass spectrometry (ICP-MS). All the polymer-SMNP complexes were small clusters with several SMNP cores incorporated together as observed by TEM (Supplementary Fig. S4).

All the polymer-SMNP complexes were stable in aqueous dispersion for over 48 hours under different ionic environments (deionized (DI) water, phosphate buffered saline (PBS), and complete media) (Supplementary Fig. 5S). The saturation magnetization values of all the clusters were in the 60–70 A m^2^/kg Fe_3_O_4_ range (Supplementary Fig. S6). Preliminary cytotoxicity studies showed that all tested polymer-SMNP complexes were minimally toxic in MDA-MB-231, BT474 and MCF10A cells at all tested concentrations (Supplementary Fig. S7).

**Figure 1:**
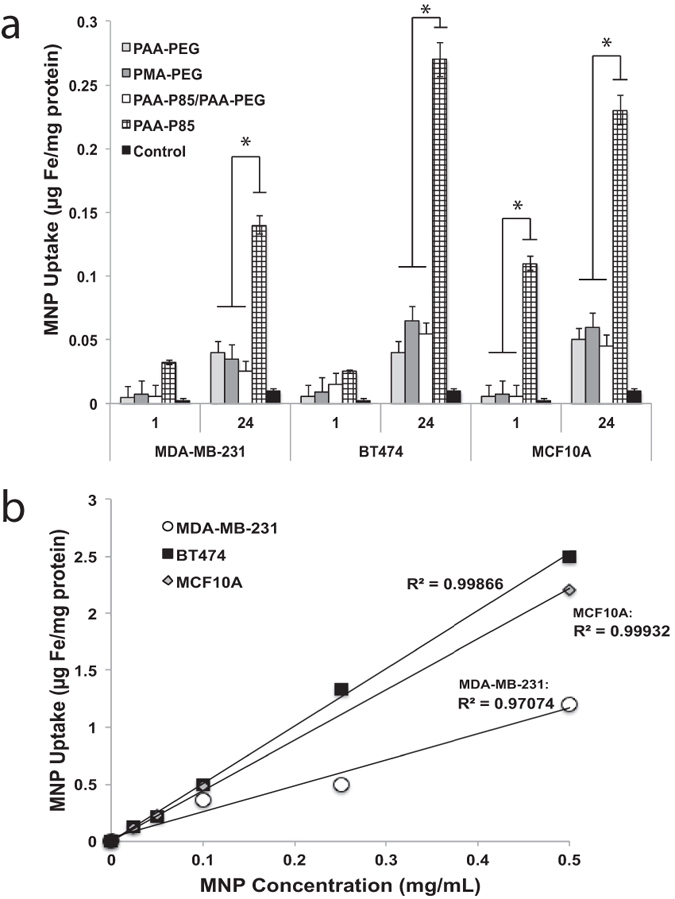
Intracellular uptake of polymer-SMNP complexes in MDA-MB-231, BT474 and MCF10 A cells. (**a**) Uptake of the polymer-SMNP complexes after incubation with complexes for 1 h or 24 h (**b**) dose dependent uptake of PAA-P85-SMNP in all three cell lines (*p < 0.05).

Internalization of the polymer-SMNP complexes was evaluated following 1 h and 24 h of incubation and was determined by the amount of Fe/mg protein in the cells (Fig. 1a). All polymer-SMNP complexes showed time and concentration dependent uptake in all experimental cell lines. PMA-PEG-SMNP showed slightly enhanced uptake compared to PAA-PEG-SMNP, especially in BT474 cells but these differences were not statistically significant. Incorporation of P85, with its relatively hydrophobic central block, into the polymer chain effectively promoted internalization of the PAA-P85-SMNPs. Interestingly, this effect of PAA-P85 was lost when PAA-P85 was mixed with PAA-PEG in the PAA-PEG/PAA-P85 blend coated SMNP. Comparable accumulation of PAA-P85-SMNP was observed in BT474 and MCF10A cells after 24 h while uptake in MDA-MB-231 was lower (Fig. 1b). Due to significantly higher uptake the following studies focused exclusively on the PAA-P85-SMNP complexes.

### Intracellular Distribution of PAA-P85-SMNP

**Figure 2:**
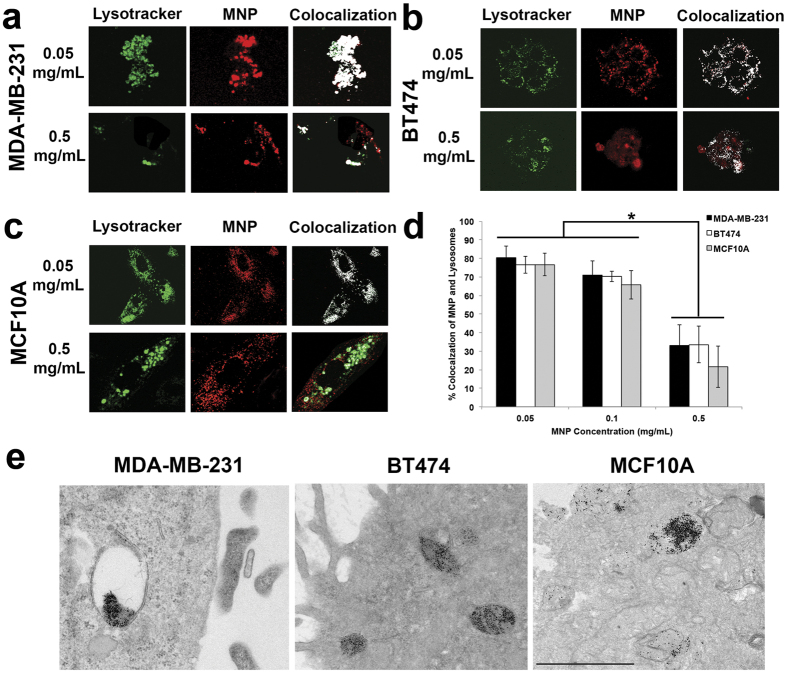
Intracellular distributions of PAA-P85-SMNPs in (**a**) MDA-MB-231(**b**), BT474 and (**c**) MCF10 A cells after 24 h of incubation with 0.05 or 0.5 mg/mL PAA-P85-SMNPs.(**d**) The quantification of the colocalization of Alexa Fluor^**®**^647-PAA-P85-SMNPs with lysosomes as determined by ImageJ/Fiji. (p < 0.01). Lysosomal encapsulation of SMNPs seen in (**e**) TEM images.

Intracellular distributions of the PAA-P85-SMNP complexes were studied by confocal microscopy in MDA-MB-231, BT474 and MCF10A cells. For this experiment, the nuclei were labeled with DAPI (blue), lysosomes were labeled with Lysotracker Green and the PAA-P85-SMNPs were labeled with Alexa Fluor^**®**^647 (red). The overlap of the Lysotracker and SMNP labels indicates colocalization. Our preliminary studies suggested that the intracellular localization of the PAA-P85-SMNPs varied at different dose concentrations. Therefore, this study dosed with both a low (0.05 mg/mL or 0.1 mg/mL) and high (0.5 mg/mL) concentration. Figure 2(a–c) shows representative confocal images of intracellular distributions of Alexa Fluor^**®**^647-PAA-P85-SMNP complexes following incubation for 24 h. As can be seen at the low concentration of 0.05 mg/mL, PAA-P85-SMNP complexes are accumulated in lysosomes, while at the high concentration of 0.5 mg/mL the PAA-P85-SMNPs also spread throughout the cytoplasm. These observations are further confirmed by the colocalization quantitative data shown in Fig. 2d. This data shows that in all three cell lines colocalization of the polymer-SMNP complexes with lysosomes remains quite high (80%) at low exposure concentrations of 0.05 and 0.1 mg/mL, but drops off significantly to about 30% at the high exposure concentration of 0.5 mg/mL. Figure 2e shows TEM data of PAA-P85-SMNPs in cells to further confirm high amounts of lysosomal accumulation.

### *In Vitro* Exposure to Super Low Frequency AC Field

Following incubation with various concentrations of PAA-P85-SMNPs for 24 h, the cells were exposed to a super low frequency AC magnetic field (50 Hz) with field strengths of 50 or 100 kA/m utilizing two exposure regimes termed ‘continuous’ (30 min) or ‘pulsed’ (10 min on, 5 min off, total 30 min on).

**Figure 3:**
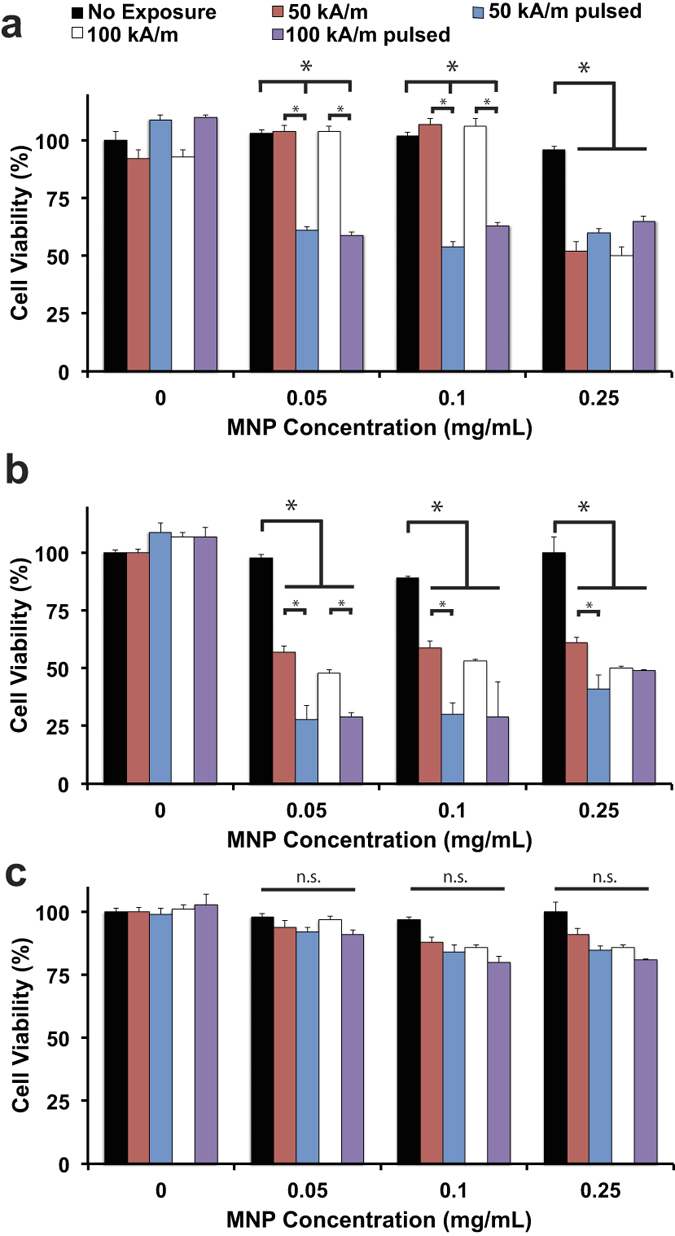
Effect of exposure to 50 Hz AC magnetic fields on cell viability. Cells were incubated with various concentrations of PAA-P85 SMNPs for 24 h, washed with acid saline and exposed to the field. Viability of MDA-MB-231 (**a**), BT474 (**b**) and MCF10A (**c**) cells was assessed following exposure to a 50 kA/m, 50 Hz or 100 kA/m, 50 Hz AC magnetic field. For each of the field strengths, two different exposure regimes were used: continuous (30 min) or pulsed (10 min on/5 min off/30 min total on) magnetic field. Data shown are mean ± SEM (n = 15), p < 0.05, n.s. = not significant.

A remarkable difference in the response of cancerous (MDA-MB-231 and BT474) versus non-cancerous (MCF10A) cells was observed. There was a significant reduction in cell viability at as low as 0.05 mg/mL of PAA-P85-SMNPs in both MDA-MB-231 (Fig. 3a) and BT474 cells (Fig. 3b) regardless of the field exposure regime utilized. However, as seen in Fig. 3c, despite similar internalization rates and SMNP concentration inside the MCF10A cells there was no noticeable decrease in cell viability after AC magnetic field exposure (all tested regimes). For the cancerous MDA-MB-231 and BT474 cells, the effect on cell viability did not occur in a dose dependent manner and was not enhanced with increased field strength. Interestingly, in the MDA-MB-231 cell line, field exposure using the continuous field regime caused little toxicity up to 0.25 mg/mL PAA-P85-SMNP complexes while in the BT474 cells, this same exposure regime caused a 50% decrease in cell viability following incubation with only 0.05 mg/mL PAA-P85-SMNP complexes. However, in both cell lines, the pulsed field regime was significantly more effective compared to the continuous field regime (50% for pulsed versus 100% cell viability for continuous field in MDA-MB-231 and 25% for pulsed versus 50% cell viability for continuous field in BT474). Exposure of the cells in the absence of PAA-P85-SMNPs to either a continuous or pulsed field regime remained minimally toxic for both cell lines. Cell viability after exposure to 0.5 mg/mL SMNPs was assessed but did not yield any higher efficacy in any of the cell lines. Due to these results, further experiments were done using a 50 kA/m field strength and the pulsed field regime.

These results show that the BT474 cells are more sensitive to the treatment than the MDA-MB-231 cells, and the healthy MCF10A cells do not seem to be affected at all. To further determine a mechanistic understanding of this observation, we first needed to determine if lysosomal membrane permeabilization (LMP) or cellular heating was responsible for the observed cell death. It has been determined, based upon our previous experimental results as well as theoretical calculations, that the observed effects cannot be explained by bulk or surface heat^16^. Previously we have clearly shown that exposure of PAA-P85-SMNP dispersions to super low frequency AC magnetic fields does not result in a temperature increase of the surrounding medium, and that changes in the physical structure of a conjugated enzyme were significantly different from a temperature-induced structural deformation^16^. Thus, we can conclude that the cell death observations are not due to heating effects.

Previous studies have indicated that exposure of cells to alternating current magnetic fields can result in mechanical disruption of the lysosomes resulting in LMP and subsequent death through these lysosomal pathways^12^,^13^,^14^. To evaluate if a similar phenomenon was occurring in our system, cells were incubated with Alexa Fluor 647-PAA-P85-SMNP and Lysotracker Green and exposed to the 50 kA/m, 50 Hz field using the pulsed field regime. Disruption of lysosomes would result in leakage of the acidic content as well as of Lysotracker Green resulting in loss of punctate fluorescence. Confocal images showed no evidence of lysosomal disruption (Supplementary Fig. S8) in any of the cell lines. Lysotracker Green remained colocalized with Alexa Fluor^**®**^647-PAA-P85-SMNPs without a noticeable decrease in Lysotracker Green fluorescence in all cell lines.

**Figure 4:**
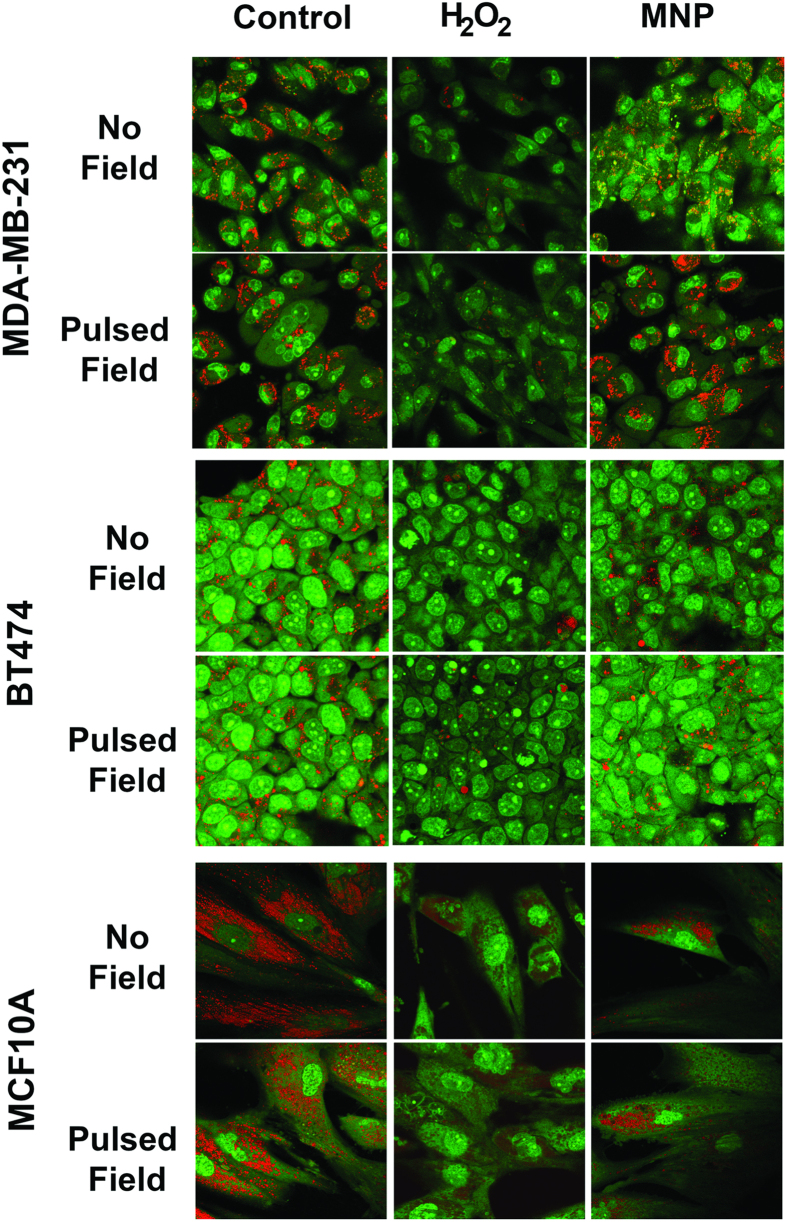
LMP detection using acridine orange in SMNP-treated MDA-MB-231, BT474 and MCF10A cells before and after pulsed field exposure. Cells were incubated with PAA-P85-SMNP for 24 h at 37 °C, washed and exposed to the 50 Hz pulsed AC magnetic field. (50 kA/m). After three hours, cells were incubated with 10 μg/mL acridine orange for 15 min. Positive control cells were treated with 150 μM hydrogen peroxide for three hours. The cells exposed to hydrogen peroxide exhibit loss of punctate red fluorescence while negative controls and cells treated with SMNPs do not.

An acridine orange assay, a more robust method to detect LMP, was also conducted. Acridine orange is a lysosomotropic stain that can be used to measure the lysosome membrane functionality. The stain is excited by UV light and emits red/orange fluorescence when in lysosomes and green fluorescence when present in the nucleus or cytosol. Cells with intact lysosomes display punctate red/orange fluorescence but this red/orange fluorescence reduces significantly after LMP^18^,^19^,^20^. Hydrogen peroxide was used as a positive control because it is known to induce LMP^21^. Figure 4 shows that SMNP incubation along with pulsed field exposure does not cause loss of lysosomal fluorescence as observed in the positive hydrogen peroxide control. The lysosomes retain the punctate red/orange fluorescence before and after field exposure in all three cell lines, which indicates a lack of LMP.

**Figure 5:**
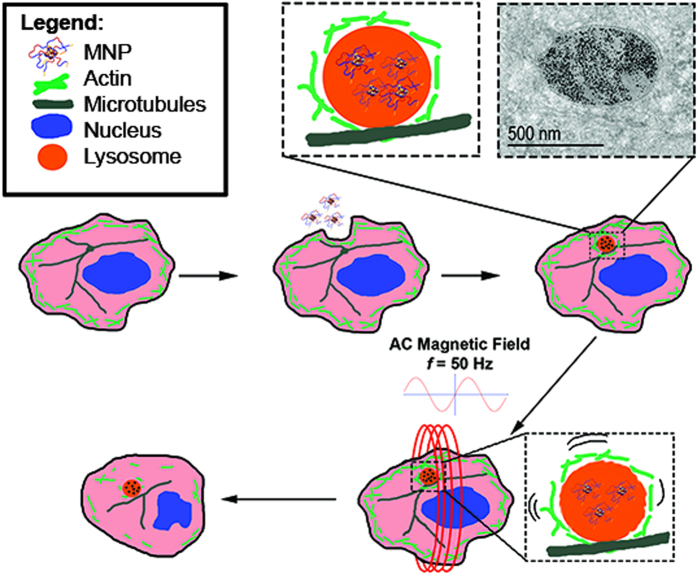
Schematic representation of SMNP uptake into lysosomes followed by mechanical movement in the lysosomes to generate forces leading to cytoskeletal disruption.

Once heating and LMP were eliminated as potential explanations for our observations, we looked to the differing cytoskeletal architectures of the cell lines for a mechanism. Cytoskeletal damage as a cause of cell death has been well reported in the literature. Actin filaments are one of the main components involved in maintaining cell structure as well as assisting with transport of organelles and vesicles throughout the cell. Previous research has shown that interference with cytoskeletal components can cause cessation of the cell cycle and lead to apoptosis^22^,^23^. Lysosomes are anchored to microtubule highways and highly associated with actin filaments. The hypothesis for this system is that the PAA-P85-SMNPs accumulate in lysosomes and upon remote actuation by the AC magnetic field can rotate inside of the lysosome, thus inducing torques and shear stresses on the underlying cytoskeleton, all without causing lysosomal leakage. A schematic of this event progression is depicted in Fig. 5. The cytoskeleton in cancerous cells is more sensitive to mechano-transduction leading to subsequent damage and cell death. Thus, it is suggested that while the generated forces are insufficient to cause damage to the underlying cytoskeleton of the stiffer, benign cells, less mechanical force is required to cause cytoskeletal deformation to the cytoskeleton of cancerous cells^24^,^25^,^26^.

**Figure 6:**
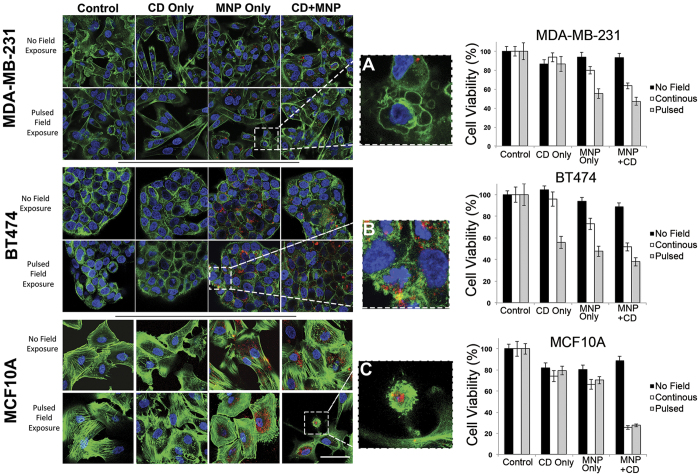
The left panel shows representative confocal images of actin (green) of the MDA-MB-231, BT474 and nontumorigenic MCF10A cells before and after exposure to a pulsed AC magnetic field with or without treatment with CD and/or PAA-P85-SMNP (red). Insets show a large image of the actin cytoskeleton of a dead. (**A**) MDA-MB-231, (**B**) BT474 and (**C**) MCF10A cell. The right panel shows corresponding cell viability for the same conditions in the three cell lines.

The theory of actin damage as the cause of cell death was studied by first determining the effect of the AC magnetic field on actin structure using confocal microscopy (Fig. 6). MDA-MB-231 and BT474 control cells show an actin filament structure very typical of cancer cells while the nontumorigenic MCF10A cells show actin structures very typical of healthy epithelial cells. Following exposure to 0.1 mg/mL of Alexa Fluor647^**®**^ labeled PAA-P85-SMNPs and a pulsed 50 Hz, 50 kA/m AC magnetic field, the confocal images revealed significant disruption of the actin cytoskeleton in the cancerous MDA-MB-231 and BT474 cells but not in the nontumorigenic MCF10A cells (Fig. 6 left panel). This is in excellent agreement with the previously discussed cytotoxicity data (Fig. 3). To further test the correlation between the mechanical properties of the cells and treatment effects, the cells were incubated with Cytochalasin D (CD). CD disrupts actin polymerization and in sub-lethal doses decreases the mechanical stiffness of cells (as measured by Atomic Force Microscopy). Therefore, exposure of non-cancerous cells to CD reduces their stiffness to the levels comparable to cancer cells^25^. Notably after exposure to CD and SMNPs the pulsed AC magnetic field regime enacts significant cytoskeletal damage in MCF10A cells as can be seen in the insert of Fig. 6c. The damage is comparable to the damage observed in the cancerous cells following exposure to the SMNPs and pulsed AC magnetic field (Fig. 6a,b). No significant differences in the cytoskeleton structure were observed in cancerous cells incubated with CD alone following exposure to a pulsed AC magnetic field.

Cell viability data confirmed these observations. Addition of CD to SMNP exposed MCF10A cells sensitizes them to both the continuous and pulsed AC magnetic field regimes (Fig. 6 right panel). The MCF10A cell viability decreased to 25% following exposure to the 50 Hz, 50 kA/m AC field. It was also interesting to see that CD appeared to sensitize the cancer cells to forces created by the SMNP in the continuous field regime.

The proposed mechanism of mechanical disruption of the cytoskeleton is in very good agreement with the differences in cytotoxicity observed for MDA-MB-231 cells versus BT474 cells. BT474 cells grow in multilayer colonies and their complex cytoskeletal structure is very important to their growth. Interestingly, we have observed colocalization of PAA-P85-SMNPs with the basal cells rather than in the top layer (Supplementary Figs S9 and S10). It may be that when the cytoskeletons of the basal cells in the colony are compromised, this causes a subsequent loss to the apical cells in the colony as well which results in the lower cell viability we observed in Fig. 3.

## Discussion

We observed a new mechanism of toxicity of SMNPs in non-heating super low frequency AC magnetic fields to cancerous cells that involves cytoskeletal disruption, and it can be selectively enacted upon cancerous cells while leaving healthy cells intact. The selective cytotoxic effect was dependent on the cell mechanical properties rather than on intracellular uptake disparities between cancerous and healthy cells reported elsewhere^14^,^15^,^27^. Notably, cancerous and non-cancerous cell lines differ in mechanical properties of the cytoskeleton. Cancerous cells are mechanically softer than their benign counterparts due to their need to remodel during transformation and metastasis^24^. For example, the Young’s moduli of malignant MDA-MB-231 cells are less than half those of non-malignant MCF10A cells^28^. It has previously been shown that SMNPs conjugated to signaling proteins can control the assembly of cytoskeletal components such as microtubules in an applied magnetic field^29^,^30^. It was also shown that SMNPs under AC magnetic fields can form linear aggregates^8^,^31^. In addition, the theory suggests that in high frequency gradient magnetic fields, SMNPs can also oscillate mechanically and produce ultrasound waves^32^. According to this theory forces generated by an assembly of SMNPs, such as those observed here in lysosomes, are sufficient to induce cellular responses^32^. However, our experiments were carried out in the absence of the magnetic field gradient, using at least 1/10 of the field amplitude and nearly 10^4^ lower field frequencies than those predicted to generate the ultrasound waves^32^. Therefore, we believe that we may observe a different effect.

We have previously reported that exposure to an AC field can cause mechanical movement of SMNPs, which generates stress forces and deformation of the surrounding polymer coating and attached biological molecules^33^. In one study, the aggregates of PAA-PEG coated SMNPs with enzymes conjugated to the PAA chains were reported to denature the enzyme in the AC field without heating^16^. The estimates of the forces for the AC fields used herein (50 Hz, 50 to 100 kA/m) suggest that the movement of individual magnetite particles of ~7–8 nm in diameter is unlikely to induce forces high enough to generate biological responses (see force estimates in the Supplementary information). However, these fields can produce forces ranging from several dozen to ~300 pN, if single particles form aggregates with a greater net magnetic moment. Such forces may exceed the strength of the filaments in the cells and result in their damage^34^. The literature states that actin-actin bonds will break at 600 pN under straight pulling and at 320 pN under twisting forces^35^. Notably, these estimates of strength were obtained with a monotonously increasing load applied to the filaments as a whole. The aggregates of the SMNPs accumulated in lysosomes can actually affect filaments locally and multiple times (~3·10^4^ loading cycles during 10 min at 50 Hz AC field). It is well known that due to fatigue dynamic strength of the materials is 3–4 times lower than their static strength.

**Figure 7:**
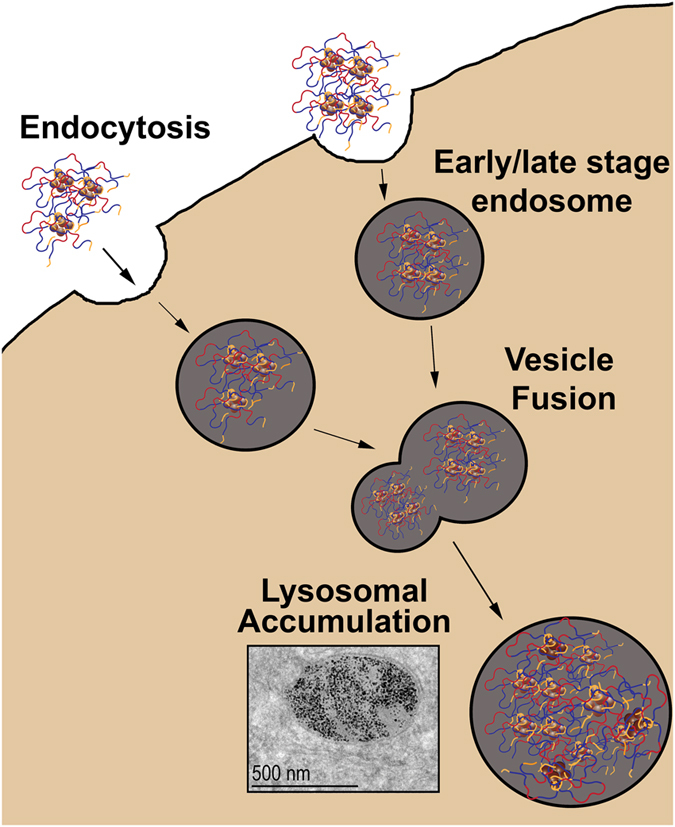
Scheme illustrating assembly of PAA-P85-SMNP during endocytosis and trafficking in the cells. The SMNP (brown spheres) are coated with the pentablock copolymer attached to the particles through the PAA chains (yellow). Upon accumulation in the lysosomes the coated SMNP form large aggregates, in which individual magnetite particles are interconnected via swollen hydrophilic poly(ethylene oxide) chains (blue) and hydrophobic poly(propylene oxide) chains (red) that interact with each other and the lysosomal membrane.

Interestingly we did not observe permeabilization of lysosomes in contrast to Zhang *et al*. who described the use of a rotating magnetic field to rotate particles, mostly within lysosomes (and attached to the lysosome membranes)^9^. Therefore, we believe that there are major differences in both mechanism and result from the work of Zhang *et al*. The present work does not invoke rotating fields and importantly the particle size ~7–8 nm is at least an order of magnitude less than that of Zhang *et al*. (they used particles of 100 nm and more). Such small particles aggregate within the lysosomes upon internalization in the cells. Each lysosome may contain from several hundred to several thousands of particles, which is not surprising since we estimate that each cell takes up from ~10^5^ to ~10^6^ particles (see Supplementary information). The pentablock copolymer coating of the particles may be very important since upon the accumulation of the particles in the lysosomes their hydrophobic poly(propylene oxide) chains of P85 may interact with each other (alike formation of Pluronic micelles). This should lead to a formation of a dense physical nanogel SMNP-polymer network. Moreover, poly(propylene oxide) chains may also form multiple “anchors” to the lysosome membrane (see ref. 36 for review of Pluronic interactions with cell membranes). As a result the giant SMNPs aggregates and the surrounding lysosomes would move as a whole with much of the load affecting their points of attachment to the actin filaments and only relatively little stress produced upon the lysosomal membranes (Fig. 7).

Notably, effects of the continuous AC magnetic field depend more specifically on the SMNP concentration inside the cells and lysosomes while exposure to the pulsed AC magnetic field generates more cell damage at each tested concentration. The exposure to CD sensitizes the cancerous cells to a continuous AC magnetic field, suggesting that less force is generated by continuous exposure. This difference between exposure to continuous and pulsed AC magnetic fields might be due to the fact that following the application of force, stress-relaxation processes can occur in the cells. Connord *et al*. reported that during the AC field application (300 kHz, 53 mT) lysosomes containing magnetite nanoparticles of nearly same size as in the present work can align within cells in needle-like structures along the direction of the field^13^. Once the field is switched off the lysosomes disassemble. Assembling and disassembling occurs during minutes, i.e. same time scale as the on/off field exposures in the present work. So it is likely that similar assembling/disassembling can also proceed in the conditions of our experiment. At lower frequencies, such 50 Hz AC field the alignment of magnetite-loaded lysosome is even more likely. In this case the rupture of the cytoskeleton may take place in the areas of greatest stress due to a combination of strain and torque. (The torque produced by AC field appears to be essential, since no toxicity was observed upon repeated exposure of cancer cells to DC field.) Once the lysosomes disassemble the strain is released, but when the field is applied again the assembly of the lysosomes may cause stress in the new areas of the cytoskeleton.

## Conclusions

Our results demonstrate that polymer coats can enhance the intracellular uptake of SMNPs and allow subsequent magneto-mechanical actuation of these nanoparticles through the use of super low frequency AC magnetic fields. The work demonstrates that cytoskeletal disruption and subsequent cell death can be selectively enacted upon cancerous cells while leaving healthy cells intact. This type of system which allows for enhanced intracellular uptake, remotely controlled actuation and most importantly cancer cell selectivity has a high impact potential for cancer therapy and could serve as a platform technology in other biomedical applications.

The Carolina Partnership, a strategic partnership between the UNC Eshelman School of Pharmacy, The University Cancer Research Fund through the Lineberger Comprehensive Cancer Center and the grant from the UNC Eshelman Institute for Innovation, in part supported this work.

## Additional Information

**How to cite this article**: Master, A. M. *et al*. Remote Actuation of Magnetic Nanoparticles For Cancer Cell Selective Treatment Through Cytoskeletal Disruption. *Sci. Rep.* **6**, 33560; doi: 10.1038/srep33560 (2016).

## Supplementary Material

Supplementary Information
