# Author Correction: Remote Actuation of Magnetic Nanoparticles For Cancer Cell Selective Treatment Through Cytoskeletal Disruption

**DOI:** 10.1038/s41598-022-14288-6

**Published:** 2022-06-16

**Authors:** Alyssa M. Master, Philise N. Williams, Nikorn Pothayee, Nipon Pothayee, Rui Zhang, Hemant M. Vishwasrao, Yuri I. Golovin, Judy S. Riffle, Marina Sokolsky, Alexander V. Kabanov

**Affiliations:** 1 Center for Nanotechnology in Drug Delivery, University of North Carolina, Chapel Hill, NC USA; 2 Department of Pharmaceutical Sciences, University of Nebraska Medical Center, Omaha, NE USA; 3 Macromolecules and Interfaces Institute, Virginia Polytechnic Institute and State University, Blacksburg, VA USA; 4 Nanocenter, G. R. Derzhavin Tambov State University, Tambov, 392000 Russian Federation; 5 Laboratory of Chemical Design of Bionanomaterials, Faculty of Chemistry, M. V. Lomonosov Moscow State University, Moscow, 117234 Russian Federation

Correction to: *Scientific Reports* 10.1038/srep33560, published online 20 September 2016

This Article contains errors.

In Figuer 4, the image for control BT474 cells, after pulsed field, was inadvertently duplicated from the control BT474 cells, no field, condition. A corrected version of Figure 4 and its accompanying legend appear below.

In addition, for Supplementary Figure S12, the images for MCF10A cells, field only; MDA-MB-231 cells, MNP only; and MDA-MB-231 cells, MNP and field treatment, are incorrect. A corrected version of Supplementary Figure S12 and its accompanying legend appear below.

These errors do not affect the conclusions of the Article.

**Figure 4:**
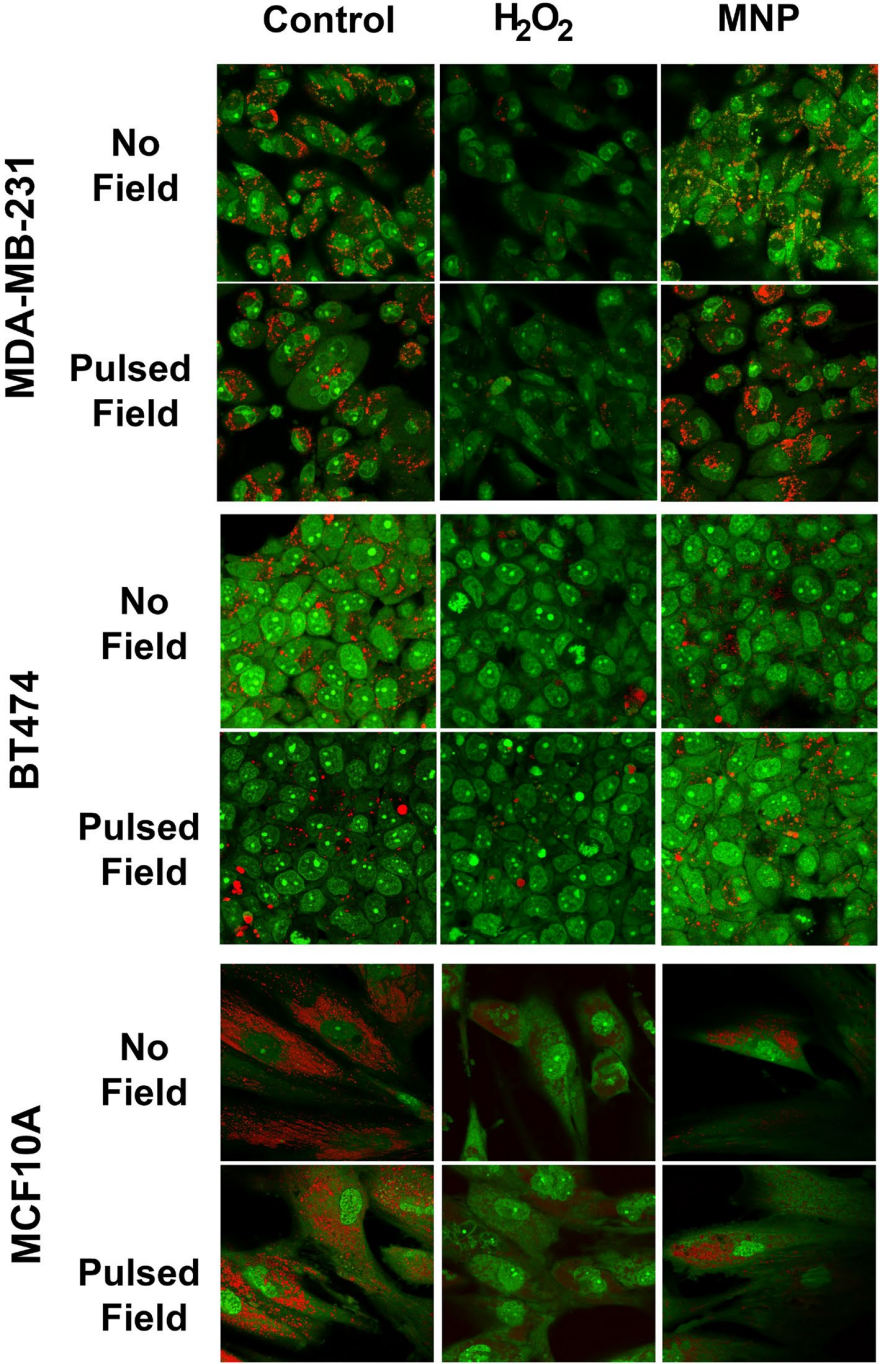
LMP detection using acridine orange in SMNP-treated MDA-MB-231, BT474 and MCF10A cells before and after pulsed field exposure. Cells were incubated with PAA-P85-SMNP for 24 h at 37 °C, washed and exposed to the 50 Hz pulsed AC magnetic field. (50 kA/m). After three hours, cells were incubated with 10 μg/mL acridine orange for 15 min. Positive control cells were treated with 150 μM hydrogen peroxide for three hours. The cells exposed to hydrogen peroxide exhibit loss of punctate red fluorescence while negative controls and cells treated with SMNPs do not.

## Supplementary Information

Supplementary Figure S12.

Supplementary Figure S12 legend.

