# Supplementary material for: Remote Actuation of Magnetic Nanoparticles For Cancer Cell Selective Treatment Through Cytoskeletal Disruption
Source: Sci Rep. 2016 Sep 20;6:33560. doi: 10.1038/srep33560 (PMC5028756; doi:10.1038/srep33560)
Supplement: Supplementary Information [file srep33560-s1.doc]

Remote Actuation of Magnetic Nanoparticles For Cancer Cell Selective Treatment Through Cytoskeletal Disruption

Alyssa M. Master,^1§^ Philise N. Williams,^1,2§^ Nikorn Pothayee,^3^ Nipon Pothayee,^3^ Rui Zhang,^3^ Hemant M. Vishwasrao,^1,2^ Yuri I. Golovin,^4,5^ Judy S. Riffle,^3^ Marina Sokolsky,^1*^ Alexander V. Kabanov^1*^

^§^ denotes co-first author

^1^ Center for Nanotechnology in Drug Delivery, University of North Carolina, Chapel Hill, NC, USA

^2^ Department of Pharmaceutical Sciences, University of Nebraska Medical Center, Omaha, NE, USA

^3^ Macromolecules and Interfaces Institute, Virginia Polytechnic Institute and State University, Blacksburg, VA, USA

^4^ Nanocenter, G. R. Derzhavin Tambov State University, Tambov, 392000, Russian Federation

^5^ Laboratory of Chemical Design of Bionanomaterials, Faculty of Chemistry, M. V. Lomonosov Moscow State University, Moscow, 117234, Russian Federation

* Corresponding Author: A.V.K., M.S. Center for Nanotechnology in Drug Delivery, UNC Eshelman School of Pharmacy, University of North Carolina at Chapel Hill, Genetic Medicine Building, Room 1094, Campus Box 7362, Chapel Hill, NC 27599-7362, Tel: +1 (919) 537-3800. E-mail: kabanov@email.unc.edu

Supplementary Information

**Supplementary Methods and Results**

**Synthesis of a PAA-*b*-P85-*b*-PAA Pentablock Copolymer (Figure S1)**


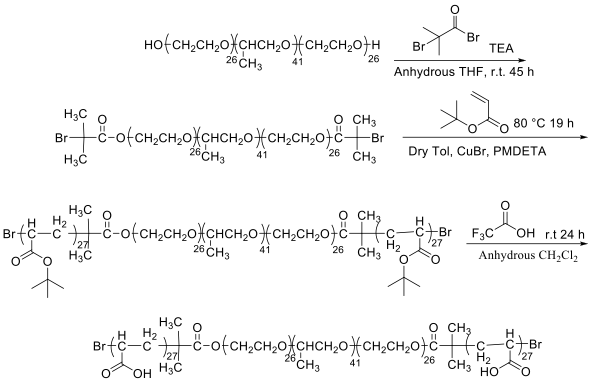


**Figure S1.** Schematic of the preparation of PAA-*b*-P85-*b*-PAA pentablock copolymer

*Synthesis of a Br-P85-Br macro-initiator*. Dihydroxyfunctional P85 was reacted with 2-bromoisobutyryl bromide to make a macro-initiator that was used for polymerization of *tert*-butyl acrylate by atom transfer free radical polymerization. P85 (9.7 g, ~2.1 x 10^-3^ mol) was dried under vacuum at 60 °C overnight, then was dissolved in anhydrous THF (100 mL) in a 250-mL round bottom flask. Triethylamine (2.3 mL, 16.5 x 10^-3^ mol) was added. The mixture was cooled in an ice bath and then 2-bromoisobutyryl bromide (2.0 mL, 16.5 x 10^-3^ mol) was added dropwise. The ice bath was removed and the mixture was stirred at room temperature for 45 h. The reaction mixture was filtered twice and THF was removed by rotary evaporation. The mixture was diluted with CH_2_Cl_2_ (110 mL) and then washed with a saturated aqueous NaCl solution twice. The organic layer was concentrated and precipitated in a 1:1 v:v mixture of chilled hexane and diethylether (800 mL each time) twice. The precipitate was filtered and dried under vacuum at 40 °C overnight.

*Synthesis of a ptBA-b-P85-b-ptBA copolymer.* Br-P85-Br was used as a macro-initiator for polymerization of *tert*-butyl acrylate. Br-P85-Br (M_n_ ~4,700 g mol^-1^, 3.0 g, ~6.0 x 10^-4^ mol), *tert*-butyl acrylate (4 mL, 2.8 x 10^-2^ mol), and dry, deoxygenated toluene (8 mL) were added into a 50-mL Schlenk flask. Oxygen was removed with three freeze-pump-thaw procedures. Cuprous bromide (0.26 g, 1.8 x 10^-3^ mol) and *N*,*N*,*N*′,*N*′′,*N*′′-pentamethyldiethylenetriamine (0.38 mL, 1.8 x 10^-3^ mol) were added quickly under nitrogen. Two additional freeze-pump-thaw procedures were applied. The Schlenk flask was sealed with parafilm and stirred at 80 °C for 19 h. After the polymerization, the reaction mixture was diluted with CH_2_Cl_2_ (60 mL). The catalyst was removed by filtering the reaction mixture through a neutral alumina column twice using CH_2_Cl_2_ as the eluent. The solution was filtered and the solvents were removed by rotary evaporation. The block copolymer was dried under vacuum at room temperature overnight.

*Deprotection of ptBA-b-P85-b-ptBA to afford PAA-b-P85-b-PAA copolymer.* The *tert*-butyl ester groups were selectively removed by a previously reported procedure using trifluoroacetic acid (TFA).^1^ P*t*BA-*b*-P85-*b*-P*t*BA (2.4 g, ~2.9 x 10^-4^ mol) was dried in a 100-mL round bottom flask under vacuum at 60 °C overnight. Anhydrous CH_2_Cl_2_ (30 mL) was added to dissolve the polymer. Trifluoroacetic acid (4.3 mL, 5.6 x 10^-2^ mol) was added dropwise and the reaction mixture was stirred at room temperature for 24 h. The polymer was precipitated into chilled hexane (400 mL). The precipitated polymer was filtered and collected. The solid was then dissolved in THF (10 mL) and dialyzed against DI water (4 L) through a cellulose acetate membrane (MWCO 1,000 g mol^-1^) for 48 h. The PAA-*b*-P85-*b*-PAA copolymer was recovered by freeze-drying for 2 d. The composition by weight was measured by ^1^H NMR to have block molecular weights of PAA(1.9k)-PEO(1.1k)-PPO(2.4k)-PEO(1.1k)-PAA(1.9k).

Synthesis of Polymer-SMNP (PAA-*b*-P85-*b*-PAA-Magnetite Nanoparticle) Complexes

Synthesis of polymer-SMNP complexes utilized a similar procedure^2^ to that previously reported to synthesize complexes with magnetite and PEO-*b*-PAA. Oleic acid-coated magnetite nanoparticles (50 mg) were dispersed in anhydrous chloroform (5 mL) in a 20-mL vial. The mixture was sonicated for 10 min. Meanwhile, PAA-*b*-P85-*b*-PAA (100 mg) was charged into a separate vial equipped with a magnetic stir bar. Anhydrous DMF (5 mL) was charged to dissolve the polymer, and the mixture was sonicated for 10 min. The magnetite dispersion was added dropwise into the polymer solution while sonicating, followed by purging with N_2_ for 5 min. The reaction mixture was further sonicated for 4 h, and the water in the sonicator was changed every 30 min. The mixture was stirred at room temperature for 48 h. The mixture was precipitated into hexane (20 mL) five times. A permanent magnet was placed under the vial to attract the complex while the supernatant was decanted to remove any solvent, free oleic acid, and other residues. The remaining solid was washed with diethylether (20 mL) 3X, and the supernatant was decanted. The nanoparticles were partially dried by purging with N_2_ for 2 h at room temperature, then were dispersed in de-ionized water (10 mL) and the pH was adjusted to 7.4. The dispersion was sonicated for 20 min. It was subsequently transferred to dialysis tubing with a 12-14k MWCO, and dialyzed against de-ionized water (4 L) for 24 h. Finally the polymer-SMNP complexes were recovered by freeze-drying for 2 d. The polymer-SMNP complexes had an intensity average diameter of 80 nm with a polydispersity index (PDI) of 0.18, as measured by dynamic light scattering (DLS). The zeta potential was -59 mV.

**Labeling of P85 with Atto 647**

The mono-amine P85 was prepared as reported previously^3^. Mono-amine P85 (3.1 mg) was reacted with a 2-fold molar excess of Atto 647 *N*-hydroxysuccinimidyl ester (1 mg) in *N,N*-dimethylformamide (0.5 mL) supplemented with *N,N*-diisopropylethylamine (2 μL). The reaction mixture was incubated at room temperature for 5 d. The P85-Atto 647 conjugate was purified on a size exclusion column (LH-20) with methanol as the eluent. P85-Atto 647 conjugation was confirmed by thin layer chromatography (TLC) prior to use.

***In vitro* Uptake of P85**

**
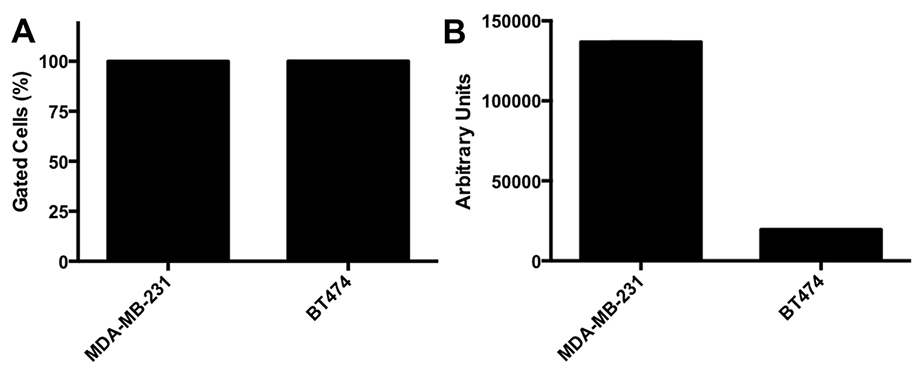
**

**Figure S2.** Flow Cytometry of P85-Atto 647**.** Cells were exposed to 0.08 ug/mL P85-Atto 647 for 1 hour, washed, trypsinized, and resuspended in PBS with 10% BSA for FACS analysis. 10,000 events were analyzed. (A) % Gated cells shows uptake into 100% of cells exposed to P85. (B) Mean fluorescence shows significant internalization of P85 into both cell lines.

*Fluorescence Activated Cell Sorting.* MDA-MB-231 and BT474 cells were seeded at 100K per well in 12 well plates and allowed to adhere for 3 d. After washing, they were treated with 200 μL of 0.08 μg/mL P85-Atto 647 for 1 h at 37 °C. This concentration is well above the CMC of P85 (6.5 x 10^-5^ M, 0.35 mg/mL). Cells were washed with PBS 3X, harvested, and resuspended in 10% Bovine Serum Albumin for FACS analysis.

*Confocal analysis on live cells.* MDA-MB-231 and BT474 cells were seeded at 20K per well in Lab-Tek II Chambered Coverglass 8-well plates. Cells were allowed to adhere for 4 d, washed and treated with 200 μL of 0.08 μg/mL P85-Atto 647, Lysotracker® and Transferrin Alexa 488 for 1 h at 37 °C. This concentration is well above the CMC of P85 (6.5 x 10^-5^ M, 0.35 mg/mL). Cells were washed 3X and kept in complete media for imaging. Live images were acquired using a Zeiss CLSM 510 LSM Confocal Laser Scanning Microscope with the 63X/oil immersion lens.

**
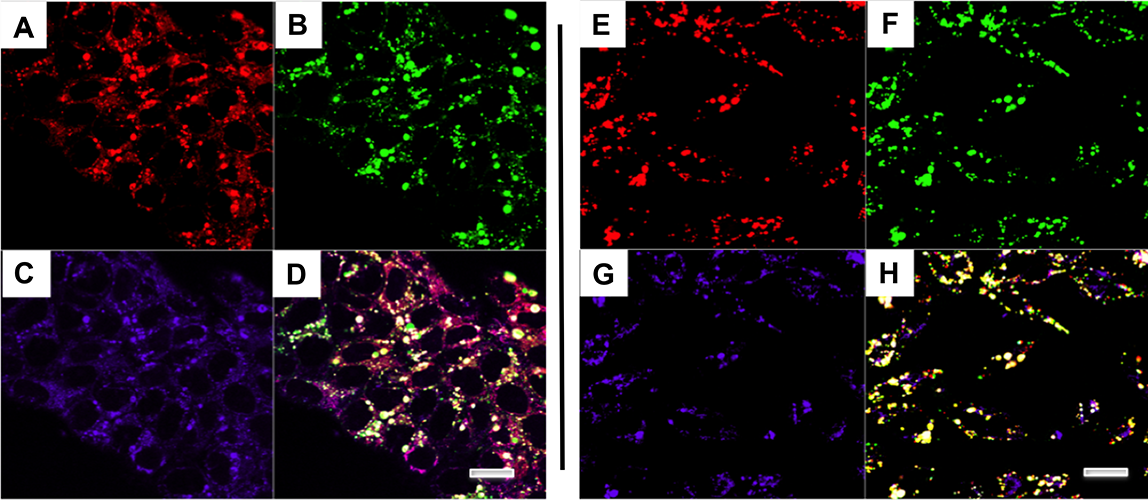
**

**Figure S3.** Confocal Microscopy of Internalized P85 in BT474 cells (left panel) and MDA-MD-231 cells (right panel). Cells were incubated with (A,E) Lysotracker Red, (B,F) 40 ug/mL Transferrin-Alexa Fluor® 488 (green), and (C,G) P85-Atto 647 1% (v/v) (purple) for 1 hour. Cells were washed and visualized by a Zeiss 510 LSM via the 63X oil immersion lens under live cell conditions. Triple colocalization is shown in the composite photo (D,H) as white punctate structures.

**Table S1. Summary of polymer-SMNP complexes used in this study**

| Polymer composition | Polymer block lengths (kDa)^a^ | Abbreviation | D_i_ (nm)^b^ | PDI^c^ | ζ-potential (mv)^d^ | Polymer content in complex by TGA  (%, w/w)^e^ | Polymer content in complex by ICP-MS  (%, w/w)^f^ |
| --- | --- | --- | --- | --- | --- | --- | --- |
| Polyacrylic acid-PEG | 7.7K-2K | PAA-PEG-SMNP | 67.0 ± 3.9 | 0.19 ± 0.01 | -39.01 ± 1.17 | 59.5 | 59.5 |
| Polymethacrylic acid-PEG | 7.2K-2K | PMA–PEG-SMNP | 55.7 ± 0.7 | 0.18 ± 0.01 | -47.03 ± 0.95 | 63.1 | 67.53 |
| 1:1 w/w blend of Polyacrylic acid-PEG and Polyacrylic acid- Pluronic P85 | 7.7K-2K/  4.6K-3K | PAA-PEG/  PAA-P85-SMNP | 38.2 ± 0.1 | 0.29 ± 0.01 | -44.23 ± 2.61 | 64.1 | 62.83 |
| Polyacrylic acid- Pluronic P85 | 1.9K-4.6K-1.9K | PAA-P85-SMNP | 30.2 ± 0.1 | 0.41±0.001 | -34.31 ± 5.2 | 65.3 | 65.32 |

^a^ Polymer block length is defined as the length of the polyacid block-length of the PEG or P85 block.

^b,c,d^ D_i_, PDI and ζ-potential were measured by DLS with Nano-ZS in de-ionized water at concentration of 0.5 mg/mL at 25^o^C. D_i_ is reported as an intensity Z-average diameter.

^c^  Polydispersity index.

^e^  Polymer content in the complex was measured by thermogravimetric analysis (TGA). Briefly, 10-15 mg samples were heated at 10^o^C/min to 110^o^C, held isothermally for 15 min and then heated at 10^o^C/min to 700^o^C.

^f^  Polymer content in the complex was measured by ICP-MS. Briefly, 1 mg/ml samples prepared, concentrated HNO_3_ (50 μL, TraCERT; Fluka) was added and the samples were incubated overnight at 70°C. The samples were then diluted to 5 mL with 2% HNO_3_, further filtered through 0.2 μm syringe filters and analyzed by Nexion 300-D ICP-MS equipped with collision cell and auto sampler (Perkin Elmer, USA). The Fe3O4 concentration was calculated based on the intensity of the 57Fe.

**TEM Images of the Clusters**

***
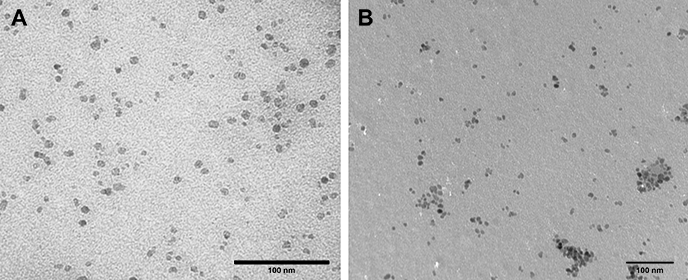
***

**Figure S4.** Representative TEM images of (A) PAA-P85 coated SMNP and (B) PAA-PEG coated SMNP.

***In vitro* Colloidal Stability of Polymer-SMNP Complexes**

Polymer-SMNP complexes were dispersed in DI water pH=6.5, PBS pH=7.4 or DMEM media (with 10 % fetal bovine serum and 1 % penicillin–streptomycin) in concentration of 1.5 mg/mL, filtered through a 0.22 μm filter and incubated at 37 ^o^C. At 1, 24 and 48 h, 0.5-mL aliquots of solution were diluted with 1 mL of the corresponding media to a final particle concentration of 0.5 mg/mL and the effective hydrodynamic diameters (D_i_) of the polymer-SMNP complexes were measured by DLS using a Zetasizer Nano ZS (Malvern Instruments Ltd., Malvern, UK). All measurements were performed in automatic mode at 25°C. All measurements were performed at least in triplicate to calculate mean values ± SD.

**
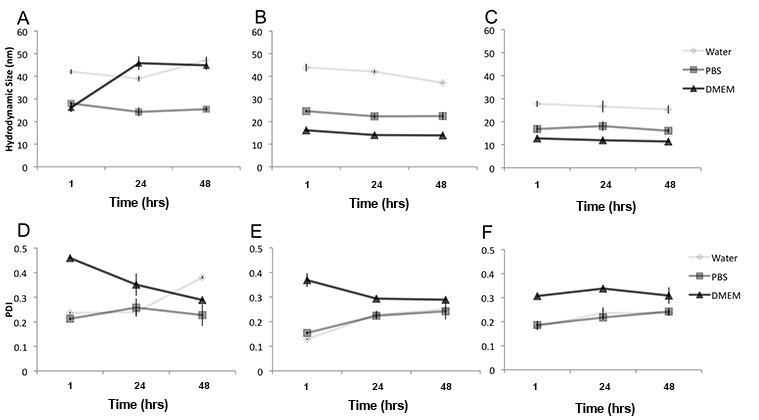
**

**Figure S5.** Particles were dispersed in solvent, sonicated, filtered at 0.22 um, allowed to stand for 45 minutes, and then measured by DLS. This graph represents three independent experiments. Row 1: Hydrodynamic intensity average diameters of (A) PAA-P85-SMNP, (B) PAA-PEG-SMNP, and (C) PMA-PEG-SMNP; Row 2: Polydispersity of (D) PAA-P85-SMNP, E) PAA-PEG-SMNP, and F) PMA-PEG-SMNP.


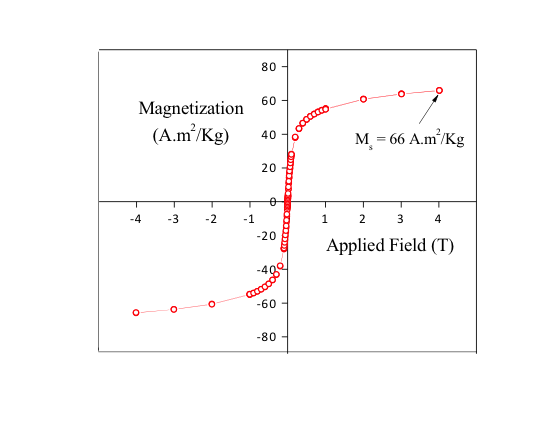


**Figure S6.** M-H (hysteresis) loop at 300 K. The saturation magnetization (M_s_) is 66 A m^2^/kg of iron oxide.

***In vitro* Cytotoxicity of Polymer-SMNP Complexes**

Polymer-SMNP complexes were assayed for cytotoxicity in the absence of AC magnetic field exposure by MTT assay as explained in the main text.

**
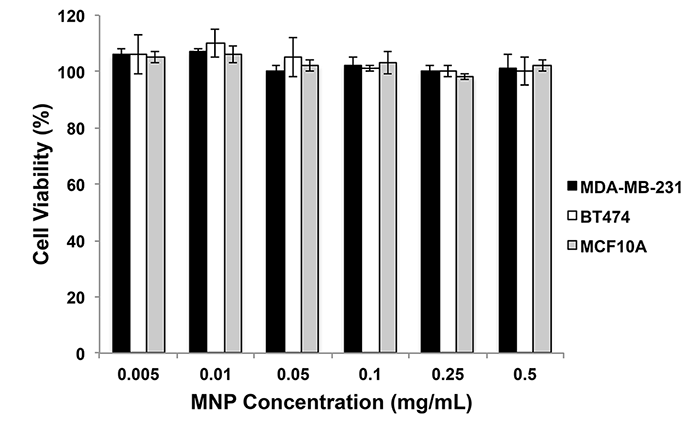
**

**Figure S7.** Cytotoxicity of polymer-SMNPs in the absence of AC magnetic field exposure in MDA-MB-231, BT474 and MCF10A cells. The cells were incubated with increasing concentrations of polymer-SMNP complexes for 24 h and washed with acid saline to remove any membrane-bound SMNP complexes. Cell viability was assessed by MTT assay 24 hours post incubation.

**Intracellular Localization of PAA-P85-SMNP**

MDA-MB-231 and BT474 cells were seeded at 20K per well in 8-well Lab-Tek II Chamber slides. Cells were allowed to adhere for 4 d and were treated with 0.05 mg/mL Alexa Fluor**®**647-PAA-P85-SMNP for 24 h. After thorough washing, cells were treated with 100 nM of Lysotracker™ Green (**λ**_ex_/**λ**_em_=504/511 nm) for 1 h. Cells were washed 3X with acid saline (pH 3) and kept in complete media for imaging. Live cell images were acquired using a Zeiss CLSM 710 Spectral Confocal Laser Scanning Microscope with the 63X/1.4 Oil Plan Apo lens. Fluorescence was quantified using Image J.


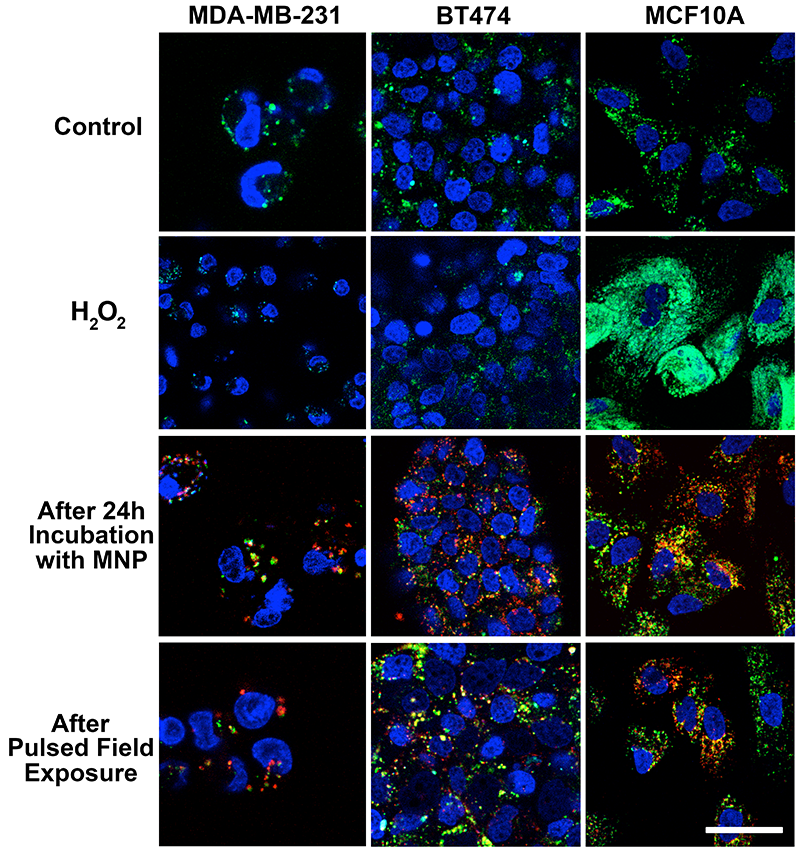


**Figure S8**: Intracellular distribution of the PAA-P85-SMNP in MDA-MB-231, BT474 and MCF10A cells before and after field exposure. Cells were incubated with Alexa Fluor 647-PAA-P85-SMNP for 24 h at 37^O^C, washed with acid saline, incubated with Lysotracker Green (Alexa 488) for 1 h and exposed to a 50kA/m, 50 Hz pulsed (10 min on/5 min off) AC magnetic field. Co-localization (yellow green) of the MNPs with the Lysotracker indicated lysosomal uptake. This figure also shows lack of lysosomal membrane permeabilization (LMP) after field exposure. The positive control (cells exposed to hydrogen peroxide) indicates Lysotracker staining after LMP. Scale bar = 20 μm

**
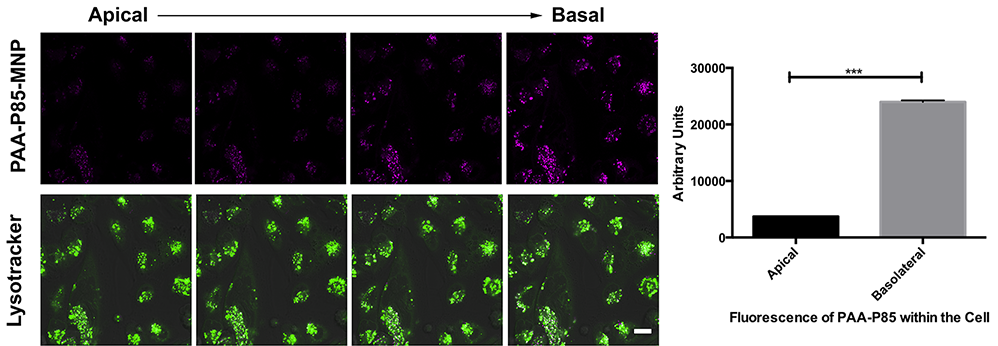
**

**Figure S9.** Confocal microscopy of MDA-MB-231 treated for 24 hours with 0.05 mg/mL AlexaFluor 647-PAA-P85-SMNP. This z-stack shows that the intracellular distribution of SMNPs increases towards the basal part of the cell. Quantification of this fluorescence is seen in the graph.

**
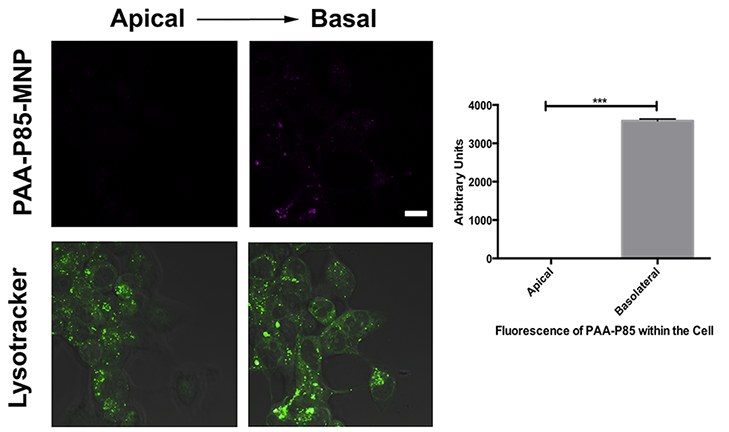
**

**Figure S10.** Confocal microscopy of BT474 cells treated for 24 h with 0.05 mg/mL AlexaFluor 647-PAA-P85-SMNP. This z-stack shows that the intracellular distribution of SMNPs increases towards the basal part of the cell. Quantification of this fluorescence is seen in the graph.

**TEM Images of SMNPs in Cells**

MCF7 cells were seeded in 6 well plates containing glass coverslips at a density of 1x10^5^ cells/well. Prior to treatment, cells were starved with incomplete media (no FBS) for 30 min. Cells were then incubated with SMNPs for 1 h at 37°C. Cells were then preserved in 4% glutaraldehyde in formaldehyde at room temperature for 24 h, then processed for TEM analysis (Figure S11).

**
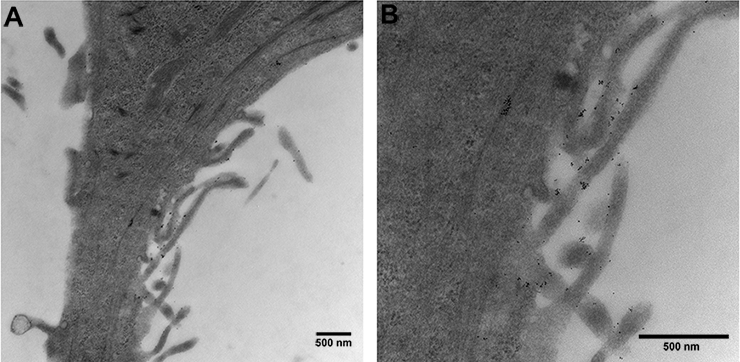
**

**Figure S11.** Representative TEM images of MCF7 cells treated with PAA-P85-SMNPs. (A) shows the association of the SMNPs with the cytoskeleton of the cells with (B) showing higher magnification.

**Mechanism of Cell Death by Flow Cytometry**

Cells were seeded in 8-well chamber slides and allowed to grow for several days. Cells were then treated with 0.1 mg/mL SMNPs for 24 h. Following incubation, cells were washed 3X with saline and then their media was replaced. Cells were then exposed to the magnetic field. For magnetic field exposure, a 50 Hz field (50 kA/m field strength) was utilized. The pulsed regime of 10 min on, 5 min off was used. Twenty-four h post-field exposure, the Annexin V/Dead Cell Apoptosis Kit with PI from Life Technologies (Carlsbad, CA) was used as per the manufacturer’s instructions. The results seen in Figure S12 corroborate data found through MTT assays. In this figure, Q1 indicates purely necrotic cells, Q2 is a mixture of late stage apoptotic cells and necrotic cells, Q3 is early stage apoptotic cells and Q4 is live cells. This further confirms that the MCF10A cells remain unaffected by the combination of SMNP and pulsed field exposure. Similarly, the MDA-MB-231 and BT474 cells yielded significant cell death after SMNP and field exposure. The figure shows that the majority of cells are in late stage apoptosis or necrosis but it is important to note that this is a snapshot of the cell death after 24 hours. Therefore, it is possible that cells that underwent apoptosis soon after field exposure may become sensitive to the PI dye by the 24-hour time point.

**
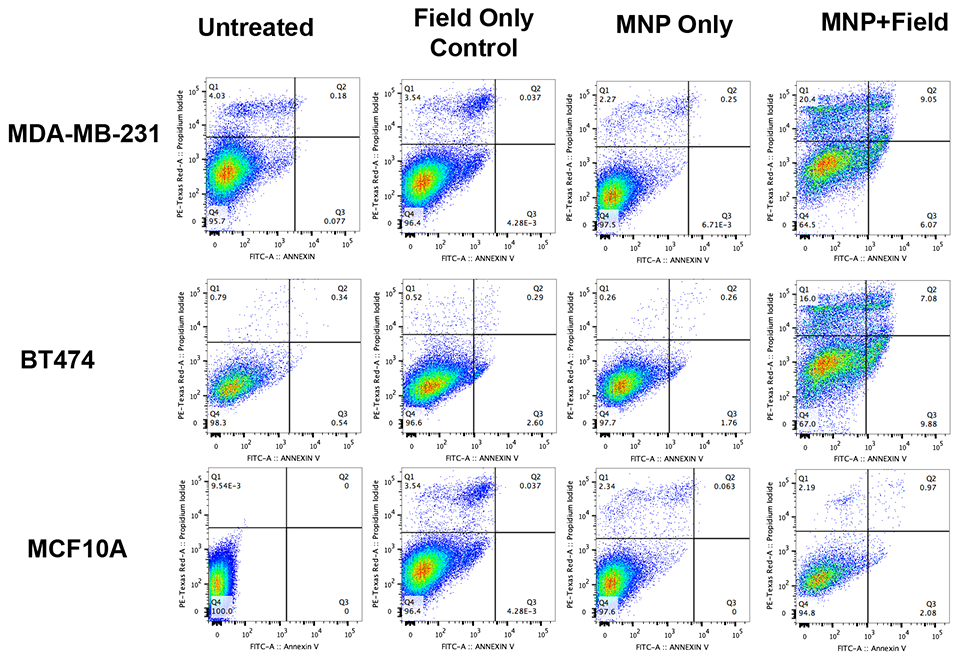
**

**Figure S12.** Results of flow cytometry assay 24 hours after pulsed field exposure. The controls of field and SMNPs only show little death. In contrast, the MDA-MB-231 and BT474 show high cell amounts of late stage apoptosis and necrosis after exposure to SMNPs and the pulsed field. The MCF10As remain unaffected by SMNP and pulsed field exposure.

**Estimate of a Number of SMNP per Cell**

Assuming magnetite SMNP take up 500 Fe ng/mg cell protein ≈ 700 Fe_3_O_4_ ng/mg cell protein and magnetite density ~ 5.2 g/cm^3^ the total volume of magnetite taken up by cells is 7·10^-7^ / 5.2 ≈ 1.34·10^-4^ cm^3^/g cell protein. The volume of one magnetite particle having a radius *R_m_* = 4 nm is approximately 4/3 x π (4 x 10^-7^)^3^ ≈ 2.7·10^-19^ cm^3^. For mammalian cells, a value for protein density of ~ 0.2 g/mL was reported (BNID 105938, see also Ref.^4^). This leads to estimates of ~ 0.5·10^15^ particles / g cell protein or ~1·10^14^ particles / mL cell volume. Assuming mammalian cell volume ranging from ~1000 to 10,000 μm^3^ (see Ref.^5^) the final estimate is from ~1·10^5^ to ~1·10^6^ magnetite particles per cell.

**Consideration of magneto-mechanical forces**

An isolated single magnetite SMNP of 7 to 8 nm in diameter (as used in our study) can produce only a relatively small force upon the surrounding, in which this particle is immobilized. The spin reorientation of the magnetic moments **µ** of such particles randomly oriented relative to the external field ***В*** (Neel relaxation) proceeds faster compared to the particle physical rotation (Brown relaxation)^6-8^. However, in the case of strong magnetocrystalline anisotropy and relatively weak magnetic fields the Neel relaxation may be incomplete and particle may undergo slower relaxation by rotation. Moreover, upon particle aggregation the magnetic moments may partially loose motility and Neel relaxation may slow down as is indirectly supported by a decrease of specific absorption rate (SAR) of SMNP aggregates in magnetic hyperthermia^7,9^. The maximal force *F_M_* that can be generated by a single Fe_3_O_4_ SMNP with a radius *R_m_* = 4 nm in a field *В* = 100 mT (root-mean value) can be estimated as follows^10-13^:

*F_M_  ≈* 1.4 µ *В/ R_HD_*  = 1.4 *J_s_ V_M_* ρ *B/ R_HD_ ≈* 3 pN

where *J_s_  =* 82 A m^2^ /kg - saturation magnetization of the magnetite, *V_M_ =* (4/3) π *R_m_*^3^ – volume of the magnetic core, ρ = 5,200 kg/m^3^ magnetite density, *R_HD_*  = 6 nm particle hydrodynamic radius (assuming that the particle is coated by a polymer). This force is insufficient to destruct a filament.

However, assuming that SMNPs aggregate (in our case due to self-assembly of pentablock copolymer chains attached to the particles and intracellular uptake, trafficking and sorting of the particles into lysosomes) the magnetic moment can increase to **µ**·*N*·*α,* where 0 < α < 1 - is a coefficient that depends on the extent of magnetic ordering of magnetic moments in the aggregate and *N* – is the number of particles in the aggregate. In this case the force *F_MA_* that such aggregate can produce would be expressed as follows:

*F_MA_  =* µ *N α B/R_HD_**

where *R_HD_** ≈ *R_HD_* (*N/k*)^1/3^ – is the hydrodynamic radius of the aggregate and *k* – the coefficient of filing of the aggregate by the particles (*k* ≈ 0.74 for a densely packed aggregate). A lysosome with a size ranging from 100 nm to several hundreds nanometers can incorporate from ~ 400 to several thousand or even dozens of thousand densely packed SMNPs of *R_m_* = 4 nm and *R_HD_*  = 6 nm. Assuming that *N* = 1000, α = 1, and *В* = 100 mT the force can be as high as:

*F_MA_  =* µ *N^2/3^ α B k^-1/3^*  *R_HD_* *^-1^*≈ 1.1 *α N^2/3^ F_M_* ≈ 330 pN

This force would be comparable with the strength of the filaments and can result in their damage ^14^.

**References:**

1 Pothayee, N. *et al.* Manganese graft ionomer complexes (MaGICs) for dual imaging and chemotherapy. *J Mater Chem B* **2**, 1087-1099, DOI:10.1039/c3tb21299h (2014).

2 Pothayee, N. *et al.* Magnetic Block Ionomer Complexes for Potential Dual Imaging and Therapeutic Agents. *Chemistry of Materials* **24**, 2056-2063, DOI:10.1021/cm3004062 (2012).

3 Yi, X., Batrakova, E., Banks, W. A., Vinogradov, S. & Kabanov, A. V. Protein conjugation with amphiphilic block copolymers for enhanced cellular delivery. *Bioconjug Chem* **19**, 1071-1077, DOI:10.1021/bc700443k (2008).

4 Milo, R. What is the total number of protein molecules per cell volume? A call to rethink some published values. *BioEssays : news and reviews in molecular, cellular and developmental biology* **35**, 1050-1055, DOI:10.1002/bies.201300066 (2013).

5 Hevia, D. *et al.* Cell volume and geometric parameters determination in living cells using confocal microscopy and 3D reconstruction. *Protocol exchange* **2011**, DOI:10.1038/protex.2011.272 (2011).

6 Chang, L. *et al.* The efficiency of magnetic hyperthermia and in vivo histocompatibility for human-like collagen protein-coated magnetic nanoparticles. *Int J Nanomedicine* **11**, 1175-1185, DOI:10.2147/IJN.S101741 (2016).

7 Dutz, S. & Hergt, R. Magnetic particle hyperthermia--a promising tumour therapy? *Nanotechnology* **25**, 452001, DOI:10.1088/0957-4484/25/45/452001 (2014).

8 Hergt, R., Dutz, S., Muller, R. & Zeisberger, M. Magnetic Particle Hyperthermia: Nanoparticle Magnetism and Materials Development for Cancer Therapy. *Journal of Physics: Condensed Matter* **18**, S2919–S2934 (2006).

9 Périgo, E. A. *et al.* Fundamentals and advances in magnetic hyperthermia. *Applied Physics Reviews* **2**, 041302, DOI:10.1063/1.4935688 (2015).

10 Golovin, Y. I. *et al.* A new approach to the control of biochemical reactions in a magnetic nanosuspension using a low-frequency magnetic field. *Tech. Phys. Lett.* **39**, 240-243, DOI:10.1134/S106378501303005X (2013).

11 Golovin, Y. I., Klyachko, N. L., Sokolsky-Papkov, M. & Kabanov, A. V. Single-domain magnetic nanoparticles as force generators for the nanomechanical control of biochemical reactions by low-frequency magnetic fields. *Bull. Russ. Acad. Sci. Phys.* **77**, 1350-1359, DOI:10.3103/S1062873813110130 (2013).

12 Golovin, Y. I., Gribanovskii, S. L., Golovin, D. Y., Klyachko, N. & Kabanov, A. Single-Domain Magnetic Nanoparticles in an Alternating Magnetic Field as Mediators of Local Deformation of the Surrounding Macromolecules. *Phys. Solid State* **56**, 1342-1351 (2014).

13 Golovin, Y. I. *et al.* Towards nanomedicines of the future: Remote magneto-mechanical actuation of nanomedicines by alternating magnetic fields. *J Control Release* **219**, 43-60, DOI:10.1016/j.jconrel.2015.09.038 (2015).

14 Noy, A. *Handbook of molecular force spectroscopy*. (Springer, 2008).
