# Supplementary figures and images for: Author Correction: Remote Actuation of Magnetic Nanoparticles For Cancer Cell Selective Treatment Through Cytoskeletal Disruption
Source: Sci Rep. 2022 Jun 16;12:10077. doi: 10.1038/s41598-022-14288-6 (PMC9203547; doi:10.1038/s41598-022-14288-6)

Untreated

Field Only  
Control

MNP Only

MNP+Field

MDA-MB-231

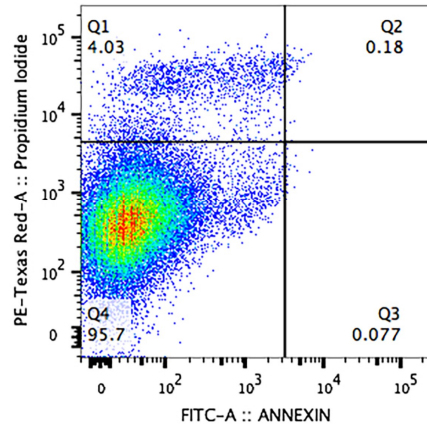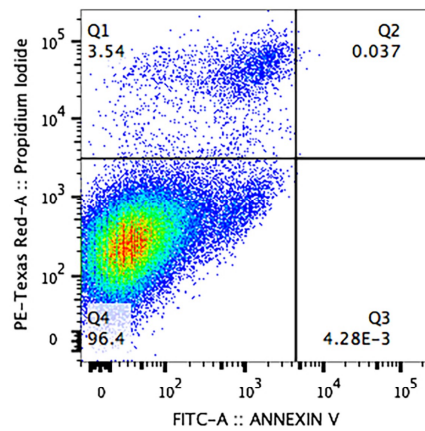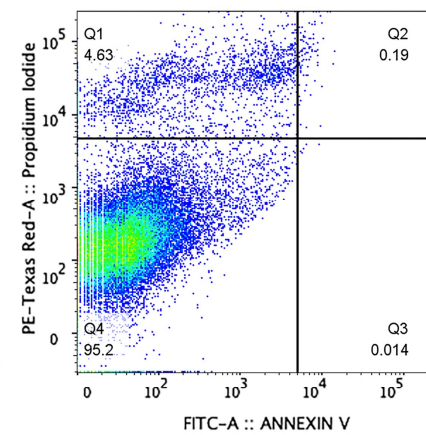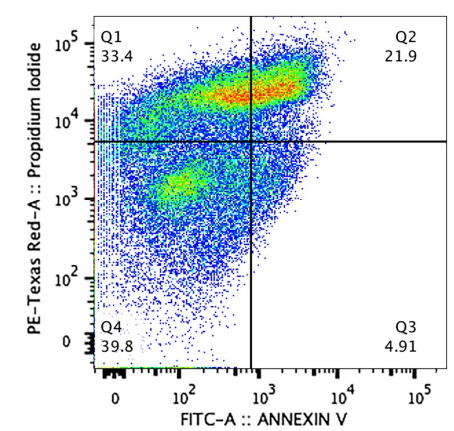

BT474

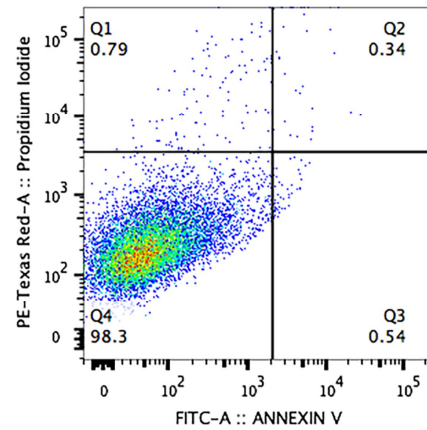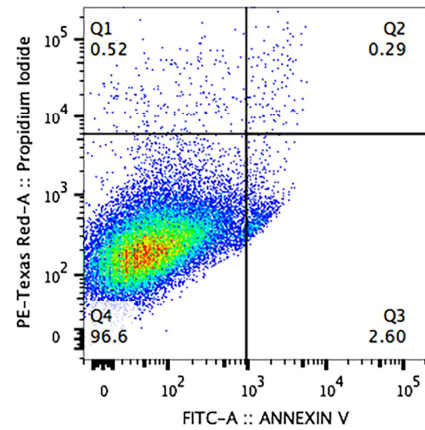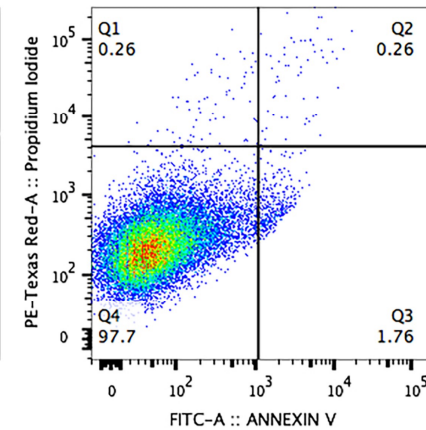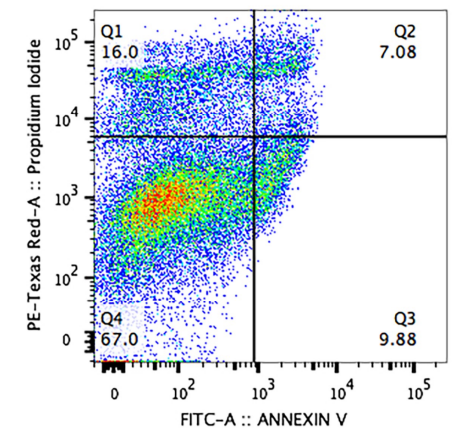

MCF10A

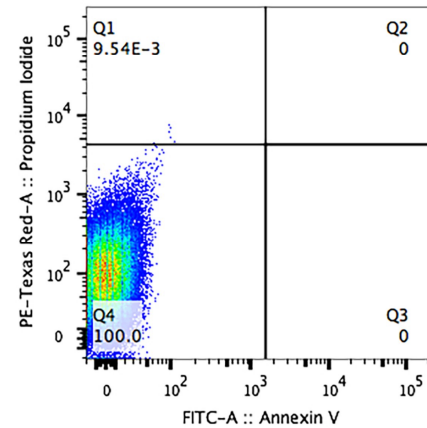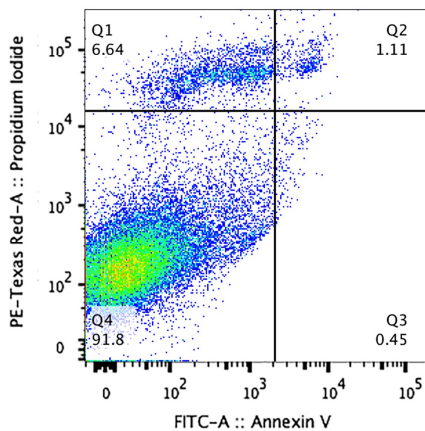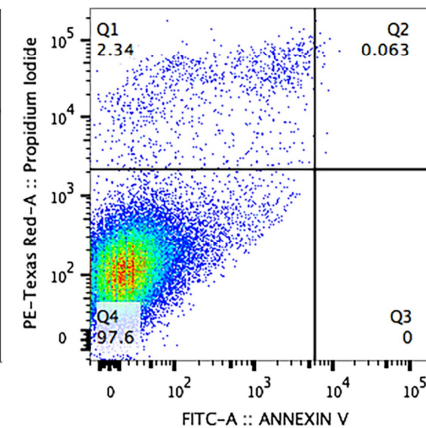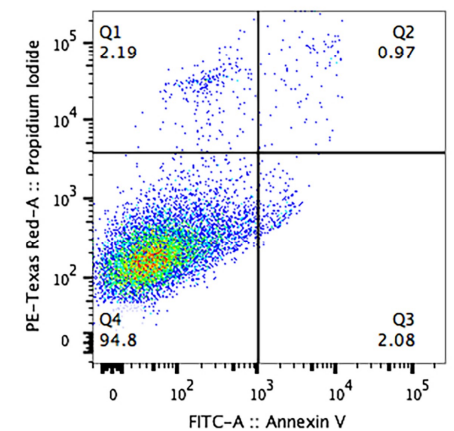

Supplement: Supplementary file 1 — Supplementary Figure S12. [file 41598_2022_14288_MOESM1_ESM.pdf]
