# Supplementary material for: Author Correction: Remote Actuation of Magnetic Nanoparticles For Cancer Cell Selective Treatment Through Cytoskeletal Disruption
Source: Sci Rep. 2022 Jun 16;12:10077. doi: 10.1038/s41598-022-14288-6 (PMC9203547; doi:10.1038/s41598-022-14288-6)
Supplement: Supplementary file 2 — Supplementary Figure S12 legend. [file 41598_2022_14288_MOESM2_ESM.docx]

Figure S12. Results of flow cytometry assay 24 hours after pulsed field exposure. The controls of field and SMNPs only show little death. In contrast, the MDA-MB-231 and BT474 show high cell amounts of late stage apoptosis and necrosis after exposure to SMNPs and the pulsed field. The MCF10As remain unaffected by SMNP and pulsed field exposure.
